# Fertilizing growth: Agricultural inputs and their effects in economic development

**DOI:** 10.1016/j.jdeveco.2017.02.007

**Published:** 2017-07

**Authors:** John W. McArthur, Gordon C. McCord

**Affiliations:** a Brookings Institution, United Nations Foundation, 1775 Massachusetts Ave NW, Washington DC 20036, United States; b School of Global Policy and Strategy, University of California, San Diego, 9500 Gilman Drive #0519, La Jolla, CA 92093, United States

**Keywords:** Agriculture, Fertilizer, Structural change, Growth, Green revolution

## Abstract

This paper estimates the role of agronomic inputs in cereal yield improvements and the consequences for countries' processes of structural change. The results suggest a clear role for fertilizer, modern seeds and water in boosting yields. We then test for respective empirical links between agricultural yields and economic growth, labor share in agriculture and non-agricultural value added per worker. The identification strategy includes a novel instrumental variable that exploits the unique economic geography of fertilizer production and transport costs to countries' agricultural heartlands. We estimate that a half ton increase in staple yields generates a 14 to 19 percent higher GDP per capita and a 4.6 to 5.6 percentage point lower labor share in agriculture five years later. The results suggest a strong role for agricultural productivity as a driver of structural change.

## 1 Introduction

Agriculture's role in the process of economic growth has framed a central question in development economics for several decades (e.g., Johnston and Mellor, 1961, Schultz, 1968). While arguments differ regarding the specific mechanisms through which agricultural productivity increases might contribute to structural change in the economy, it has long been theorized that advances in agriculture can promote shifts in labor to higher productivity sectors that offer higher real incomes. Empirical work in more recent years has helped inform the conceptual arguments, and underscored the long-term growth and poverty reduction benefits from agriculture, especially for the most extreme forms of poverty (e.g., Gollin et al., 2007, Ravallion and Chen, 2007, de Janvry and Sadoulet, 2010, Christiaensen et al., 2011). Africa's recent years of economic growth seem to follow a regular pattern of structural change away from agriculture to other economic sectors (McMillan and Harttgen, 2014).

At the same time, additional evidence underscores the role of the manufacturing sector in driving structural change and long-term convergence in incomes across countries (McMillan and Rodrik, 2011, Rodrik, 2013). The theoretical ambiguity on the role of agricultural productivity growth in structural change is captured by the formulation of Matsuyama (1992), which shows opposite effects in closed economies and small open economies. These debates and other evidence regarding agriculture's relatively low value added per worker compared to other sectors (e.g. Gollin et al., 2014) have prompted some researchers to narrow the number of developing countries in which agriculture is recommended as a priority sector for investment (Collier and Dercon, 2014). These issues present a first order concern for understanding why some countries have not experienced long-term economic progress and what to do about it. If agriculture can play a central and somewhat predictable role within the poorest countries, then it is a natural candidate for targeted public investment.

The theoretical and empirical literature regarding structural change is vast, yet statistically identifying the causal role of agricultural productivity is challenging. Indicators of structural change tend to trend together in the process of development, impacts on labor force structure are likely to occur after a lag, and the macroeconomic nature of the question is not amenable to micro-style experiments. Our contribution is to focus on the role of agricultural inputs as drivers of higher yields and subsequent economic transformation, using the unique economic geography of fertilizer production in our identification strategy. The paper builds on the insights of Lagakos and Waugh (2013), which highlights the gaps in understanding of cross-country variations in agricultural productivity. A variety of studies have estimated sources of total factor productivity (TFP) in agriculture in the poorest countries, including in sub-Saharan Africa (e.g., Bates and Block, 2013, Block, 2014), but agriculture is such an input-intensive sector that TFP assessments only provide one piece of the overarching crop sector puzzle. Our cross-country analysis also complements Bustos et al. (2016), who look at structural change in Brazilian municipalities with the diffusion of agricultural technology and practices in soy and maize.

Our econometric strategy proceeds in two parts. First, we empirically assess the inputs that contributed to increased productivity in staple agriculture, as proxied by cereal yields per hectare, during the latter decades of the twentieth century. Using cross-country panel data, this forms a macro-level physical production function for yield increases. We confirm that fertilizer, modern variety seeds, and water are key inputs to yield growth, even with aggregate data, and controlling for other factors such as human capital and land-labor ratios. Second, we construct a novel instrumental variable (IV) to examine the causal link between changes in cereal yields and aggregate economic outcomes, including gross domestic product (GDP) per capita, labor share in agriculture, and non-agricultural value added per worker. The results provide evidence that increases in cereal yields have both direct and indirect positive effects on economy-wide outcomes, and are particularly pertinent when considering economic growth prospects for countries where a majority of the labor force still works in low-productivity agriculture.

Large-scale nitrogen fertilizer production occurs in a limited number of countries around the world, owing partly to the fact that the Haber-Bosch process requires natural gas. Gravity models have demonstrated that bilateral trade declines in distance, and have been applied to study the relationship between trade and other outcomes such as wealth (Frankel and Romer, 1999) or the environment (Frankel and Rose, 2005). These papers study aggregate trade, but a similar logic can be applied to study the supply of an individual product. Given fertilizer's role as an input in agricultural production, analysis of the economic geography of fertilizer follows the findings of Redding and Venables (2004) in how “supplier access” to intermediate goods matters for trade and income per capita (see also Amiti and Cameron, 2007, Francis and Zheng, 2012). Our identification strategy exploits the global distribution of fertilizer production and subsequent transport distance to the agricultural heartland of developing countries as a source of variation in supplier access. Interacting this distance measure with temporal variation in the global fertilizer price generates an instrument for fertilizer use. To our knowledge this is the first application of economic geography towards causally identifying the relationship between agriculture and structural change.

The IV results suggest that for a country like Mali (one standard deviation above the mean distance measure), a 10% negative price shock to global fertilizer prices would increase fertilizer use by approximately 0.8 kg/ha, increase yields by 7 kg/ha (0.007 tons/ha), increase GDP per capita by 0.3%, decrease the labor share in agriculture by 0.1 percentage points over the following five years, and might increase labor productivity in the non-agricultural sector by around 0.2% over the next nine years. Meanwhile, a country with better supplier access like Jamaica (one standard deviation below the mean distance) would experience a 3.7 kg/ha increase in fertilizer use, a 34 kg/ha (0.034 tons/ha) increase in yields, a GDP per capita increase of approximately 1.3%, a decrease in labor share in agriculture by 0.3 percentage points over the next five years, and an increase in non-agricultural labor productivity of around 0.8% over the nine years (a higher annual growth rate of 0.1%).

The next section of the paper motivates the empirical work, drawing from the many contributions in the literature towards understanding structural change. Section 3 presents empirical models both for estimating the physical production function for cereal yields and for estimating the effect of yield increases on economic growth, labor share in agriculture, and non-agricultural value added per worker. Section 4 describes the data, Section 5 presents the results, and Section 6 concludes.

## 2 The green revolution and structural change

The term “green revolution” was coined following the advent of South Asia's rapid increases in cereal yields in the late 1960s, and is typically used to describe the early stage where yields jump from roughly 1 ton per hectare to 2 or more tons per hectare. Some researchers have argued that these green revolutions underpinned later stages of economic growth, and cite Africa's lack of a green revolution as a key reason why the region has not yet experienced greater long-term economic success (e.g., Diao et al., 2010).

Our empirical work draws on the long theoretical tradition on agriculture-driven structural change dating back to Rostow (1960) and Johnston and Mellor (1961). Mathematical formulations are presented by Matsuyama (1992), Laitner (2000), Hansen and Prescott (2002), Gollin et al. (2002), Gollin et al. (2007), Strulik and Weisdorf (2008), and others. In a stylized story of green revolutions, improvements in agricultural technology are achieved through the introduction of improved land management techniques and improved inputs, including germplasm and fertilizer, all of which boost yields and labor productivity (Murgai, 2001, Restuccia et al., 2008). It is important to note the historical complementarity of agronomic inputs. Modern variety seeds were very fertilizer responsive, and success was often predicated on good water management investments and broader policy support for agriculture. Our economic geography lens leads us to focus on fertilizer, since it is the part of the green revolution package most sensitive to transport costs.

This paper's empirical contribution to understanding the complexity of structural change relates the agricultural labor share directly to agricultural productivity, suggesting that satiation in food demand from Engel's law generates labor movement out of agriculture as that sector's productivity increases. If food is relatively non-tradable beyond local markets, then increased staple food production leads to reduced food prices, increased real wages and hence lower poverty. As staple yields jump and basic food needs are met, crop production begins to diversify, including to cash crops (possibly for export), and so the virtuous cycle of commercial farming begins. With greater savings and access to finance, farms begin to substitute capital for labor, and freed up workers begin to look for wage employment, typically in nearby cities. To the extent that other sectors enjoy higher labor productivity, this is welfare enhancing.

It is also possible (and we test this empirically) that agricultural productivity improvements trigger further increases in non-agricultural labor productivity. One potential mechanism is that after subsistence needs are surpassed, savings rates increase, and the subsequent capital accumulation increases worker productivity (Lewis, 1954). In parallel, governments are able to collect revenues to finance growth-enhancing infrastructure, such as roads and ports, which increases worker productivity in manufacturing and services. Kuznets (1968) summarizes four main channels through which agricultural growth contributes to economic growth: a forward linkage effect (agriculture providing food and raw materials to non-agricultural production), a backward linkage effect (agriculture consuming industrial products such as insecticide or tractors), inter-sectoral transfers (agriculture contributes taxes and cheap labor to other sectors), and foreign exchange (through agricultural exports). Another mechanism may be that increased farmer incomes improve health outcomes, thus increasing worker productivity, decreasing child mortality, reducing total fertility rates, increasing investment per child, and decreasing demographic pressures. Or, it may simply be that the non-agricultural sector enjoys increasing returns to scale due to fixed costs or learning-by-doing,^1^ which would imply that a green revolution and the resulting labor shift would accelerate productivity growth in that other sector. Although our paper is not able to pinpoint which of these mechanisms is at work, our contribution is to provide a causal framework for evaluating whether higher staple yields trigger labor shifts away from agriculture and faster growth in non-agricultural labor productivity.

### 2.1 Motivating evidence

**Fig. 1:**
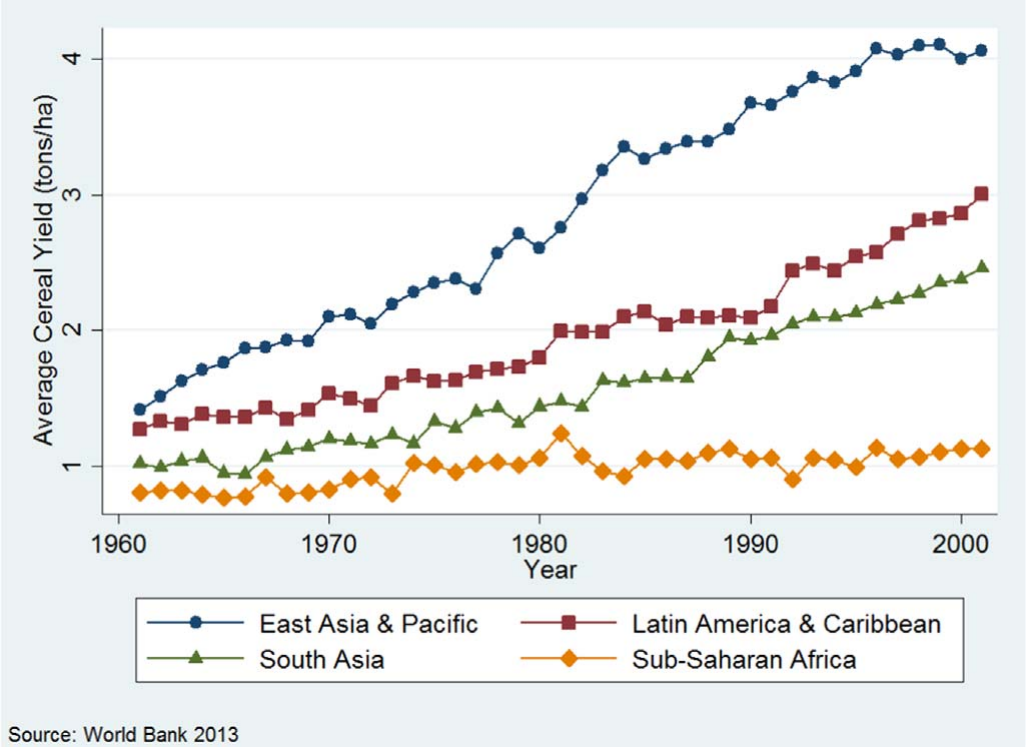
Cereal yields across developing regions, 1961–2001.

Trends in the data support the link between staple crop yields and economic growth. Fig. 1 presents cereal yields per hectare from 1961–2001.^2^ All developing regions except Africa experienced major sustained growth rates in land productivity over the period, despite varying starting points, and all except Africa more than doubled yields by 2001.

**Fig. 2:**
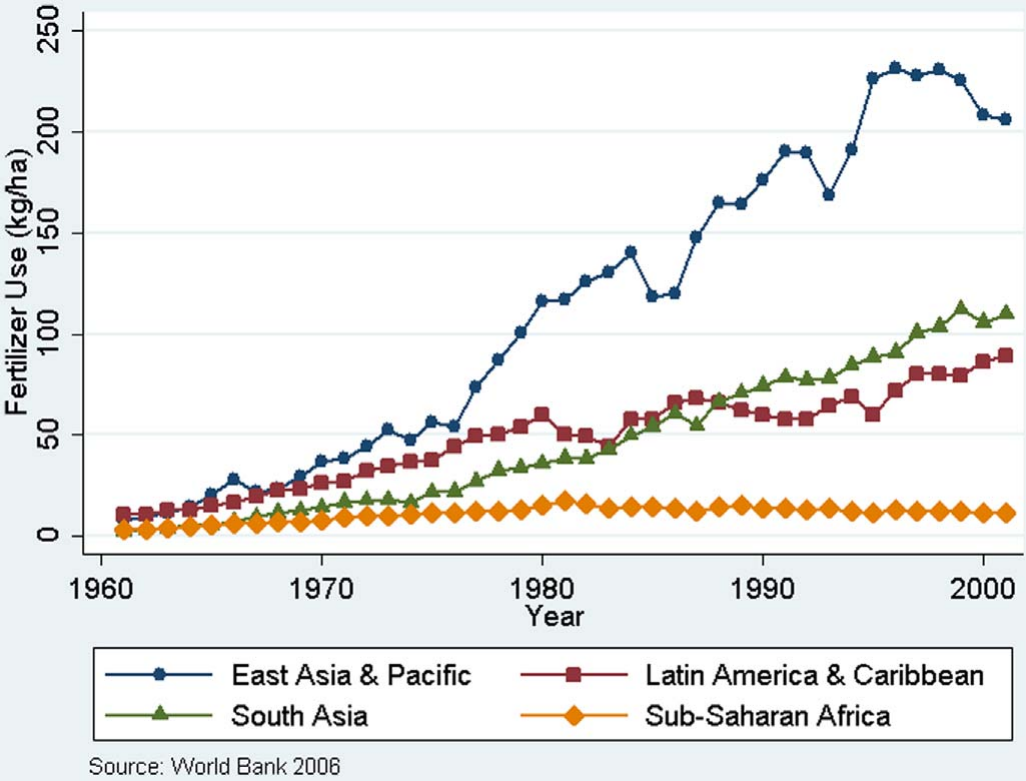
Fertilizer use in developing regions, 1961–2001.

A simple Boserup (1965) hypothesis would argue that Africa's yield stagnation relative to other regions is a product of its land abundance, and yields will increase as land becomes scarce. There are three main reasons why this hypothesis does not hold (McArthur, 2013). First, the history of 20th century yield take-offs in the developing world was predominantly characterized by proactive public policies supporting a package of yield-boosting inputs, rather than by factor scarcity (Djurfeldt et al., 2005). These policies are thought to explain much of the regional variations in fertilizer use since 1960, as shown in Fig. 2. Second, labor/land ratios vary tremendously across Africa but they are just as high or higher in many African countries than they were in pre-Green Revolution Asian countries. Third, land productivity is driven by the crucial latent variable of soil nutrients, which are being depleted at dramatic rates throughout Africa. This rapid depletion strongly suggests that land pressures in many countries are not being neutralized by extensification.

Fig. A.1 in the Appendix compares the growth of cereal yields to growth in GDP per capita over the 1965 to 2001 period, exhibiting a positive correlation of 0.44. A noteworthy point emerges from comparing 1965 cereal yield levels to subsequent 1965–2001 GDP growth; no country in the sample^3^ experienced negative average growth after reaching a yield threshold of 2 tons/ha as of the beginning of the period (see Fig. A.2 in the Appendix). The relationship between cereal yield growth and growth in non-agricultural value added per non-agricultural worker is similar to that in Fig. A.1, suggesting that higher rates of progress in agricultural productivity are structurally correlated with higher growth rates in non-agricultural labor productivity.

## 3 Empirical model

The first part of our empirical strategy establishes a country-level physical production function for cereal yields in order to motivate the emphasis on agronomic inputs in a study of structural change. We introduce our instrument in a first-stage regression for fertilizer use, and show that the fitted value for fertilizer use is a significant determinant of yields in the second stage. The second part of the empirical strategy deploys our instrument (now for yields in the first stage) to identify the causal impact of increased yields on economic outcomes and structural change as measured by GDP per capita, labor shares, and non-agricultural value added per worker.

### 3.1 Cereal yield production functions

The agricultural production function analysis builds on previous insights dating back to Hayami and Ruttan (1970) and more recent analyses reviewed in Eberhardt and Teal (2013). We apply panel data to identify a cross-country cereal yield production function, using the following baseline fixed effects specification:

(1) $$a_{\mathrm{it}}=\beta_{0}+\beta_{1}f_{\mathrm{it}}+\delta \prime X_{\mathrm{it}}+\eta_{t}^{a}+\varepsilon_{\mathrm{it}}^{a}$$

$$\varepsilon_{\mathrm{it}}^{a}=\mu_{i}^{a}+\nu_{\mathrm{it}}^{a}$$

where *a*_*it*_ is the average cereal yield per hectare in country *i* in year *t*; *f* is the average fertilizer use per hectare; and X is a vector of controls including precipitation over a calendar year, the share of seeds that are modern varieties, labor inputs, the share of arable land that is irrigated, average years of schooling as a measure of human capital, and physical machinery per hectare. Meanwhile *η*_*t*_ is a time period dummy to flexibly capture global trends, μ_*i*_ is a country fixed effect and *ν*_*it*_ is a random error term. The *a* superscript indicates a parameter specific to the agricultural production equation, distinct from the economic growth equations below.

**Fig. 3:**
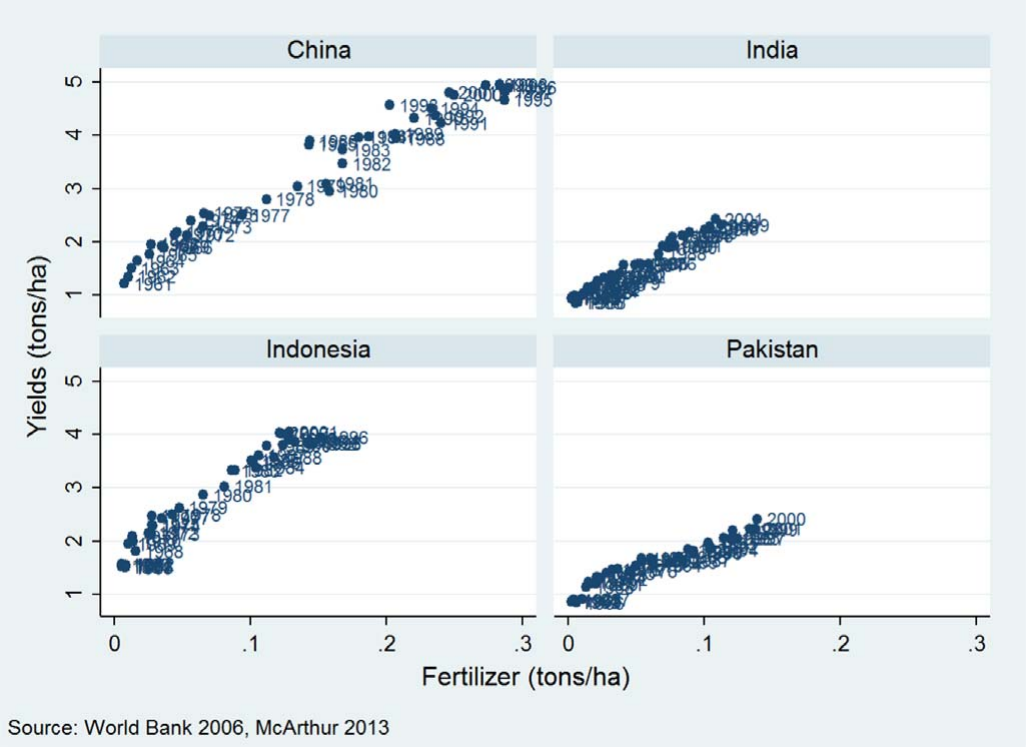
Cereal yields and fertilizer use, selected countries, 1961–2001.

The linear approximation strategy is not without limitations. It was chosen over log-linear and log-log approaches since neither of those were found to provide a better fit with the data, and indeed most countries with significant input use have pursued relatively linear fertilizer-yield trajectories, as shown in Fig. 3. This linear relationship is somewhat at odds with the field-level agronomic data that show decreasing returns, but is likely an inherently limited product of the country-level unit of aggregation. We will show that our results are robust to excluding outliers, a potential concern when using levels specifications, and for completeness the robustness section presents results using log specifications instead.

#### 3.1.1 Instrumenting for fertilizer use

**Fig. 4:**
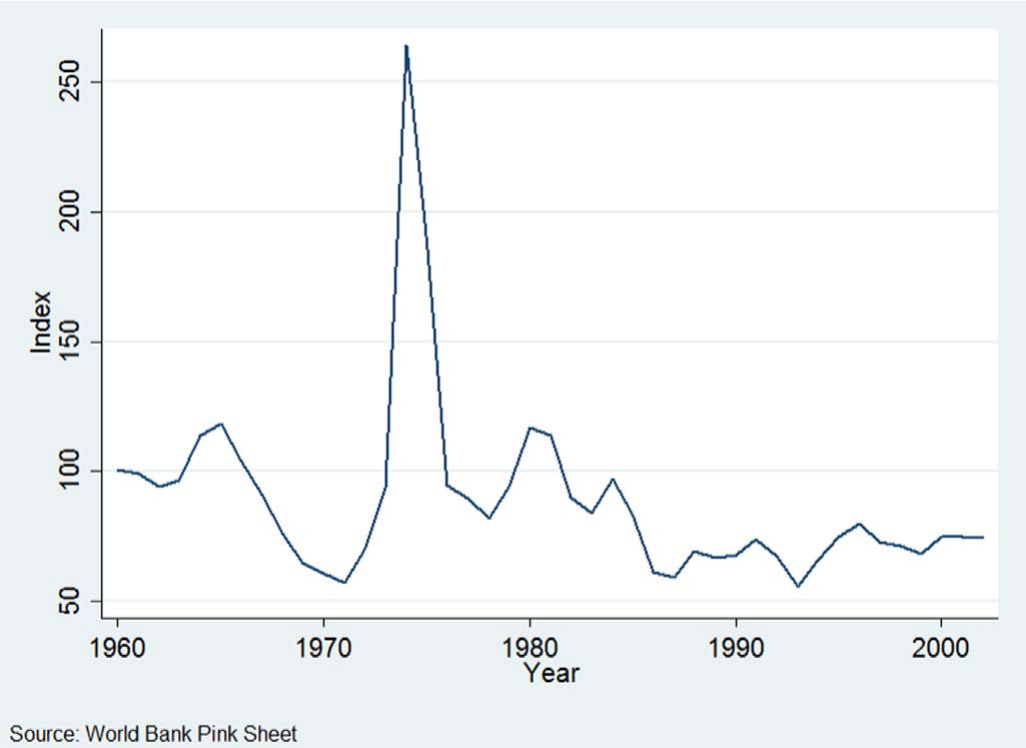
Fertilizer price index (Real US$ terms, 2005=100).

One might hesitate to interpret associations between agronomic inputs and yields as causal. Omitted variables such as farmers' agronomic know-how might be correlated with both yields and inputs and thus bias coefficients in the estimation. In addition, there is likely significant measurement error in the country-level aggregates for yields and fertilizer use (Jerven, 2010), which would lead to attenuation bias (an underestimation of the impact of increasing fertilizer use on yields). In order to assuage these concerns and improve identification, we construct a new time-varying instrument for fertilizer use. Our approach follows a similar motivation to the instrument presented in Werker et al. (2009). A valid instrument needs to be correlated with countries' fertilizer use and satisfy the exclusion restriction of not affecting yields through any channel besides fertilizer use. We use fluctuations in the global fertilizer price to generate temporal variation exogenous to conditions in any one developing country. In order to generate the cross-sectional variation in the instrument we exploit the fact that the production of nitrogen fertilizer is intensive in natural gas and therefore produced in only a select group of facilities around the world, most (but not all) of which are in developed countries. This deep advantage in fertilizer production generates a specific economic geography that endows countries with different supplier access to this intermediate good for agricultural production. Following the tradition of gravity models of trade, this varying distance to fertilizer production generates exogenous variation in trade volumes and fertilizer consumption. We contend that the distance fertilizer travels from these facilities to the agricultural heartlands of each developing country is a valid source of exogenous cross-sectional variation. Specifically, we hypothesize that countries closer to fertilizer plants are more sensitive to the commodity's price variation relative to the transport costs that farmers incur. We calculate each country's average cost-distance to the nearest fertilizer production site, weighting each grid cell within a country by the percentage of the cell planted to staple crops. We then interact the global fertilizer price with the inverse of each country's cost-distance to the nearest fertilizer production site to generate a valid time-series instrument for fertilizer use in developing countries. The fertilizer price index is constructed by the World Bank (2012), and is a weighted average of the prices of natural phosphate rock, phosphate, potassium and nitrogenous products.^4^ The index is constructed in real US dollar terms, and set to 100 for a base year (2005 in our data). Fig. 4 plots the evolution of the price index during the years in our sample. With the exception of the price spike in the mid-1970s due to the increase in prices of petrochemical products during the Arab oil embargo, fertilizer prices generally declined, especially in the years since 1980. Note that our results are unchanged if we exclude the years of the fertilizer price spike (1974–1975).

The instrument satisfies reverse causality concerns (small emerging economies are unlikely to influence global fertilizer price) and the omitted variable bias concern is assuaged since a problematic omitted variable would need be to correlated with the global fertilizer price and have the same distance decay function from agricultural heartlands to global fertilizer production facilities. In particular, the instrument would be problematic if the distance function we used reflected connectivity of countries to global markets, since it would then confound variations in global market conditions with distance to each country's manufacturing sectors, for example. However, our distance decay function has endpoints different from those for connectivity to global markets: agricultural-area weighted averages will be in different locations from population and manufacturing centers, and the nitrogen fertilizer locations are not all in the developed world nor necessarily where large economic markets are located.

A specific concern regarding global markets is variation in global food prices: if global fertilizer prices are correlated to global food prices and if our distance decay function is inadvertently capturing access to global markets, then our empirical results could be generated by changes in global food prices leading to changes in agricultural production decisions in developing countries. We test whether our results are robust to changes in global food prices and countries' access to global markets by interacting the global price with an exogenous measure of trade access from Frankel and Romer (1999). The trade access is constructed by regressing 1985 trade shares on distance between trading partners and aggregating to the country level to produce fitted trade shares for each country as a function of distances to other markets. We interact these exogenous trade shares with the global food price, such that countries with higher trade shares face higher proportional variation in the local food price as a function of global prices. Our results are robust to this test, suggesting that our instrument is not inappropriately measuring access to global markets.

Another specific concern that a reader might have is that fertilizer price fluctuations might be correlated to fossil fuel prices, which might affect economic outcomes through many channels. However, the correlations between crude oil prices and phosphate, DAP, urea and potash prices are only between 0.11 and 0.38 over the period (World Bank, 2012). Moreover, the correlation is only problematic if the specific distance decay function we use from agricultural areas to nitrogen facilities matches the pattern of cross-country differences in fossil fuel prices, and there is no reason to believe that this will be the case. We will show that all results in this paper are robust to controlling for global crude oil price interacted with the inverse agriculturally-weighted distance to fertilizer production sites.

**Fig. 5:**
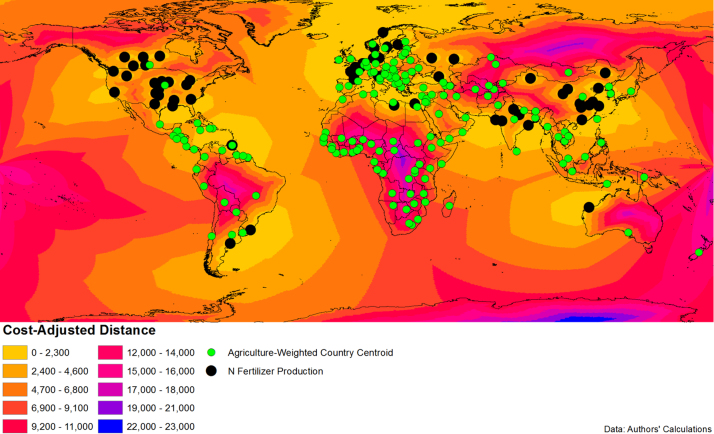
Cost-adjusted distance to major fertilizer production site.

To construct the instrument, we use a Geographic Information System (GIS) to map the percentage of each 5 arc-minute grid cell's area planted to staple crops (maize, wheat, rice, sorghum or millet), based on Monfreda et al. (2008). Next, we used Wikipedia to identify the ten largest ammonia fertilizer producers by volume (Agrium, CF Industries, EuroChem, IFFCO, Koch, Orascom Construction Industries, Potash Corporation of Saskatchewan, Sinopec, TogliattiAzot, and Yara International), and used their websites and annual reports to find the locations of their fertilizer production facilities (data on ammonia-specific facilities are not available, so we use all fertilizer facilities for these producers). We were able to identify 63 unique locations around the world where these companies produce fertilizer, and used Wikipedia to find the geographic coordinates. The countries we designate as fertilizer exporters produce around 55% of the fertilizer consumed by the countries in our sample.^5^ We then calculated the minimum cost-adjusted distance from each grid cell within a country to the nearest fertilizer production site, and calculate an average for each country weighting each grid cell by its area planted to staple crops. In order to adjust for relative transport cost between land and water, we use the result of Limão and Venables (2001) that shipping a standard 40-foot container from Baltimore to different destinations around the world in 1990 cost $190 for an extra 1,000 km by sea and $1,380 for an extra 1,000 km by land. This indicates roughly a 1:7 cost ratio, which we use to optimize travel over sea and navigable rivers versus travel over land. The fertilizer production sites and optimal cost-distance function are mapped in Fig. 5, together with each country's agriculturally-weighted centroid to give the reader a sense of where agricultural areas are located.

The locations of the fertilizer production facilities are listed in Table A.1 in the Appendix. Although these are present-day facilities, and ideally we would have beginning-of-period facilities to assuage endogenous location concerns, most facilities are located in developed countries which are not included in our sample and many are located in proximity to natural gas deposits. Readers might be concerned that development of gas deposits might be correlated to a country's level of development, thus making the distribution of fertilizer production sites endogenous to economic variables. However, fossil fuel discovery and exploitation occurs across the entire spectrum of development levels, typically conducted by major international firms, so observed natural gas exploitation is likely indicative of the exogenous distribution of natural deposits. Another concern is that fertilizer production locates predominantly in response to market demand, and our measure would therefore be picking up endogenous market conditions in fertilizer producing countries. As we discuss in Section 5.3 below, our core results remain consistent after excluding developing countries that have fertilizer production within them, suggesting that identification is coming from the cross-country relative distances to fertilizer production among non-fertilizer producers.

**Fig. 6:**
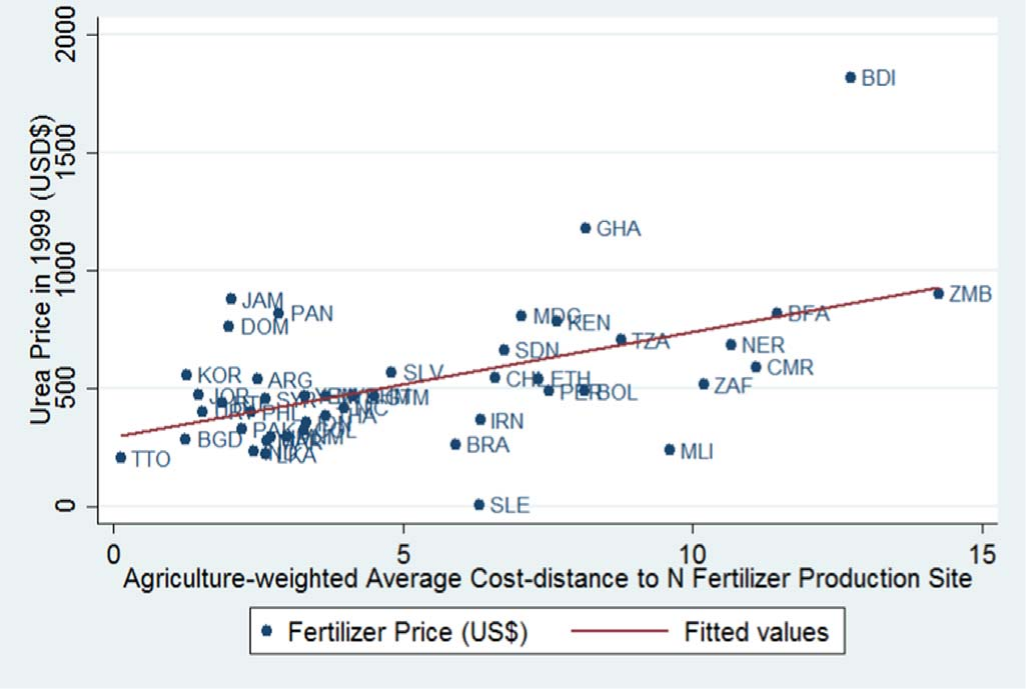
Fertilizer prices in 1999 and distance to fertilizer production site.

**Fig. 7:**
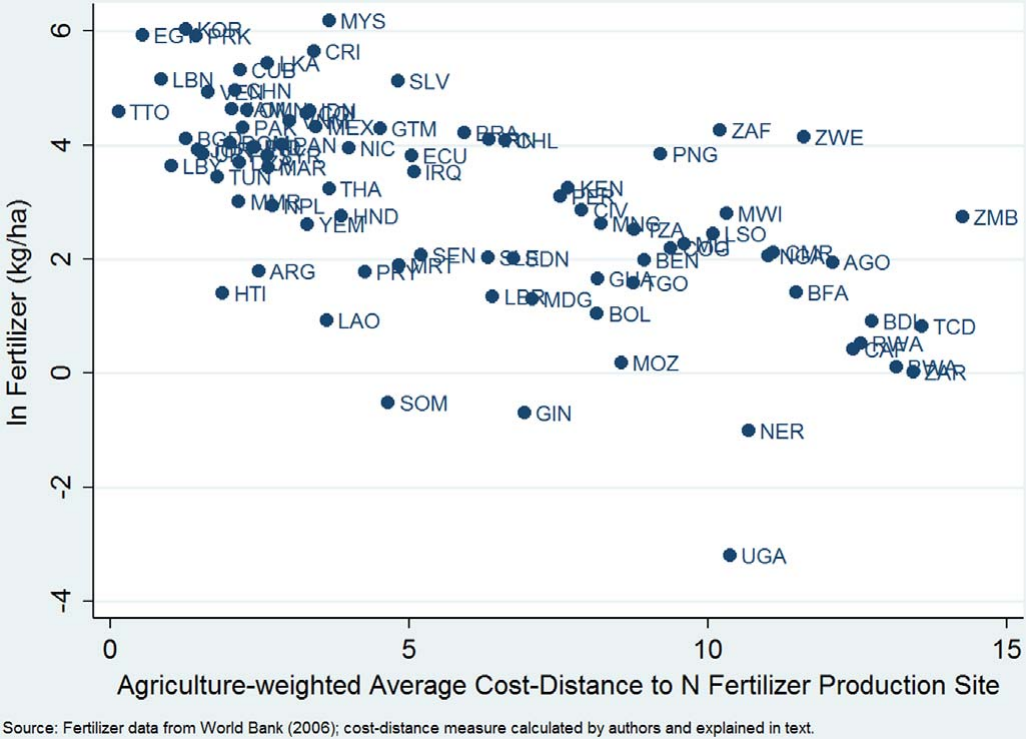
Fertilizer use in 1985 and distance to fertilizer production site.

To check whether the distance measure is capturing something about fertilizer transport costs facing developing countries, we collected fertilizer price data from FAOSTAT's Fertilizers Archive. Reported prices are those paid by farmers in local currency per metric ton, with subsidies deducted wherever possible. The most complete data for prices are available for 54 countries in 1999, so we convert those to US$ using average 1999 exchange rates from the World Development Indicators.^6^ Given that our identification strategy partially rests on the idea that natural gas production is required for nitrogen fertilizer production, we use the price of urea (the most nitrogen-rich fertilizer in common use) and in Fig. 6 plot prices against the cost-distance function we use for the instrument. Countries with smaller cost-distance to fertilizer production sites are paying lower prices on average. India (IND) has a distance index value of 2.41 and a urea price of $232 per ton (around one standard deviation below the sample mean of $526), while Burkina Faso (BFA) has a distance index value of 11.47 and a urea price of $819 per ton (one standard deviation above the mean). The correlation between price and distance is 0.52, and the regression line shown has a t-statistic greater than 3, whether or not high-price outlier Burundi is included. To test whether nitrogen fertilizer production locates near natural gas sites, we compare each country's distance index to natural gas deposits and to fertilizer production sites and find a correlation of 0.76. A third correlation of interest is between the distance component of the instrument and fertilizer use across countries. Fig. 7 investigates this by plotting the log of fertilizer use per hectare at the 1985 sample midpoint against the indexed distance measure. The correlation between the two is −0.63. Towards the top left of the scatter plot, Lebanon (LBN) has a distance index value of 0.84 and uses 175 kg/ha of fertilizer on average, while Burundi (BDI), towards the bottom right, has a distance value of 12.73 and uses 2.5 kg/ha of fertilizer.

The instrument allows us to employ the following two-stage least squares specification (using the vector X to summarize other covariates discussed above):

(2) $$f_{\mathrm{it}}=\alpha_{0}+\alpha_{1}{\mathrm{IV}}_{\mathrm{it}}+\delta 'X_{\mathrm{it}}+\varphi_{t}^{a}+\xi_{i}^{a}+\pi_{\mathrm{it}}^{a}$$

ait=β0+β1f^it+θ′Xit+ηta+μia+νita

*β*_1_ is now estimated using the fitted value of fertilizer use *f* from the first regression, and better identified in a causal sense compared to Eq. (1) above. Here *φ*, *ξ*, and *π* represent time dummies, country fixed effects, and a random error term, respectively.

### 3.2 Economic growth and labor share equations

It is trivial for higher agricultural productivity to be linked to higher economic growth in the same period, since agricultural output is included directly in national accounts. For example, if one holds fixed all prices and production levels in other sectors, a green revolution-style five year doubling of output in a low-income country with 30 percent of GDP in food production would translate mechanically to a 5.4 percent annual real GDP growth rate.^7^ For a country with only 15 percent of GDP in food production, the same yield doubling would translate to 2.8 percent annual growth. Of course a major supply expansion would be expected to decrease the price of food and the nominal measured growth rate might be much smaller.

Beyond this mechanical relation between yields and growth, however, the arguments of Sachs et al. (2004) and McArthur and Sachs (forthcoming) posit that increasing agricultural yields in low-income settings creates scope for structural change via increased savings, investment, and TFP as food becomes cheaper and minimum subsistence requirements are met. This hypothesis can be examined a few different ways. First, a cross-country growth equation for GDP per capita captures the mechanical element of agricultural-to-GDP growth plus indirect aspects of increased investment and higher TFP. Second, a cross-country framework can examine the extent to which increases in staple crop productivity trigger labor movement out of agriculture. Third, a cross-country equation for non-agricultural value added per non-agricultural worker can look for signs of more indirect pathways of investment and TFP increase in non-agricultural sectors.

The baseline fixed effect specification is constructed as follows:

(3) $$\ln\left(y_{\mathrm{it}}\right)=\rho \ln\left(y_{i,t-5}\right)+\lambda_{0}+\lambda_{1}a_{i,\mathrm{lag}\;t}+\lambda_{2}k_{i,t-5}+\lambda_{3}r_{i,t-5}\;+\omega \prime {\mathrm{MAC}}_{i,t-5}+\eta_{t}^{y}+\varepsilon_{\mathrm{it}}^{y}$$

$$\varepsilon_{\mathrm{it}}^{y}=\mu_{i}^{y}+\nu_{\mathrm{it}}^{y}$$

In Eq. (3), *y* represents average real GDP per capita in the first set of specifications and non-agricultural value added per worker in subsequent specifications; $a_{i,\mathrm{lag}\;t}$ is cereal yield per hectare in previous years (the lag structure will be discussed below); $k_{i,t-5}$ is lagged aggregate physical capital per worker; $r_{i,t-5}$ is the total fertility rate as a proxy for demographic pressures and capital widening; ${\mathrm{MAC}}_{i,t-5}$ represents a vector of standard macroeconomic variables used in the growth literature, averaged from years t−5 to t; and the *y* superscript indicates a parameter specific to the growth equation. The main coefficient of interest is *λ*_1_. Since the regression controls for country-specific effects, growth over the previous period, and initial income per capita within the period, a significant and positive value for *λ*_1_ would lend support to the importance of agricultural land productivity in boosting economic growth. As with the yield regression, we use the instrument described above to improve identification of the causal impact of changes in cereal yield. We have earlier established that we have a valid instrument for fertilizer use; by extension it is valid for yields as well. Instrumenting for yields reduces concerns of reverse causality or omitted variables bias.

Note that there is no ex-ante expectation whether *λ*_1_ is biased upwards or downwards in the absence of an IV. If, for example, there is an omitted variable such as a pro-rural government policy that distorts markets to help farmers at the expense of the overall economy (such as price floors on agricultural products), then the policy could generate an association between higher yields and slower overall economic growth, biasing *λ*_1_ downwards. On the other hand, urban-led economic development leading to higher incomes, higher demand for food, higher food prices, higher profits for farmers, and thus more investment in agronomic inputs and higher yields would indicate reverse causality from economic development indicators to yield growth. This would bias *λ*_1_ upwards. Instrumenting for yields also resolves attenuation bias from measurement errors of a coarse variable such as country-level staple yields per hectare.^8^

Fixed effects estimators suffer from dynamic panel bias particularly pertaining to bias on the lagged dependent variable (Wooldridge, 2002, Bond, 2002). As part of our robustness exercises presented in the Appendix, we conduct a complementary estimation strategy for the economic growth equations through the use of Arellano and Bond's (1991) generalized method of moments (GMM) “difference” estimator, which purges the fixed effects.

The specification we employ to study the effect of yield increases on labor share in agriculture follows the same logic as Eq. (3). However, since the share of employment in agriculture is a bounded variable, we do not estimate a dynamic model including a lag of the dependent variable as we do in the GDP or NAVA regressions. The other independent variables, including the instrumented version of cereal yields, remain the same.

## 4 Data

The estimation strategy draws upon a cross-country panel data set constructed for developing countries over the period 1961–2001. Most of the variables are constructed in 5-year intervals over the period from 1965–2000, based on data availability. Descriptive statistics are in Table 1, and a description and source of each variable is listed in Table A.2 in the Appendix. Much of the data comes from the World Bank's World Development Indicators (WDI), including cereal yield per hectare, fertilizer use per hectare,^9^ share of agricultural land under irrigation, and tractors per hectare. A new fertilizer measurement protocol was implemented after 2002, so that is the most recent year that can be included in a relevant time series, as reported in WDI 2006. The key cereal yield variable is defined as follows in the WDI: “kilograms per hectare of harvested land, and includes wheat, rice, maize, barley, oats, rye millet, sorghum, buckwheat and mixed grains. Production data on cereal yields relate to crops harvested for dry grain only. Cereal crops harvested for hay or harvested green for food, feed, or silage and those used for grazing are excluded.” (World Bank, 2006). The data count double cropping as part of an annual yield measure rather than counting only the yield per harvest.

**Table 1:** Descriptive statistics for core sample.

	**All periods**		**1965**		**2000**	
**Variable**	**Mean**	**N**	**Mean**	**N**	**Mean**	**N**
Cereal Yield (tons/ha)	1.63	588	1.18	73	2.11	75
	(1.02)		(0.60)		(1.34)	
						
Fertilizer (tons/ha)	0.059	588	0.023	73	0.091	75
	(0.10)		(.040)		(0.14)	
						
Precipitation (mm)	1278	588	1276	73	1242	75
	(820)		(792)		(809)	
						
Modern seeds (%)	13.2	554	0.30	69	28.2	70
	(19.5)		(0.99)		(26.2)	
						
Labor-land ratio	0.56	588	0.49	73	0.62	75
(thousands per sq. km.)	(0.72)		(0.65)		(0.78)	
Irrigation (%)	13.2	568	10.0	68	15.8	74
	(17.9)		(15.5)		(20.1)	
						
Years of Schooling	3.44	544	2.00	68	5.11	68
	(2.24)		(1.47)		(2.36)	
						
Tractors per ha	0.0058	487	0.0032	72	0.0123	45
	(0.0090)		(.0051)		(0.0192)	
						
GDP per capita	2640	559	1924	62	3277	73
(constant 2005 $)	(2546)		(1656)		(3367)	
Investment	19.5	499	15.5	54	21.1	71
(% of GDP)	(7.9)		(6.1)		(8.5)	
Inflation	60.2	359	–	0	17.7	64
	(387.9)		–		(38.9)	
						
Government Consumption	13.5	485	11.4	53	13.1	71
(% of GDP)	(6.2)		(6.2)		(6.6)	
Total fertility rate	5.34	588	6.42	73	4.06	75
	(1.65)		(0.98)		(1.60)	
						
Labor Share in Agriculture	58.5	588	68.3	73	49.6	75
	(23.9)		(20.0)		(25.5)	
						
Non-agricultural Value Added per worker (constant 2005 $)	5705	439	6661	33	5065	69
	(4749)		(5129)		(4806)	
Global Fertilizer Price Index	94.7	588				
	(36.9)					
						
Cost-Distance to Nearest Fertilizer Production Site	5.78	588				
	(3.75)					

*Note*: Values given for sample of 5-year intervals from 1965–2000.

Standard deviations indicated in parentheses.

Human capital is estimated by Barro and Lee's (2013) measure of total years of schooling. Values of real GDP per capita in constant 2005 US dollars are taken from Version 7.1 of the Penn World Tables (Heston et al., 2012). The labor share in agriculture is calculated using agricultural labor force size data from the FAOSTAT database. This is merged with World Bank (2013) data on cereal area planted in order to calculate the labor to land ratio variable. The numerator and denominator are not a perfect match in this instance, particularly when non-food cash crops represent a large share of agriculture, nonetheless the variable proxies for population pressures on land.

The cereal yield production functions include a historical measure of the introduction of green revolution technology from Evenson and Gollin (2003) and previously presented in Conley et al. (2007). The indicator describes modern variety (MV) crops planted as a percentage of all crops planted, weighted by area planted to those crops. The development of modern seed varieties suitable to Africa's unique crop mix and agroecological zones lagged behind the development of high yield varieties relevant to other regions by roughly two decades (Evenson and Gollin, 2003), so this variable captures the highly relevant proliferation of MVs across countries. Data for the variable cover 85 countries from 1960 to 2000, taken in five-year averages.

Monthly gridded precipitation data are taken from the University of Delaware (Matsuura and Willmott, 2012). Values are summed for each year and averaged over the country, and then converted to natural log form. This is an imperfect signal, since it is rain variability during the location-specific crop growing season that matters most, rather than precipitation across an entire year. Constructing such a location-specific precipitation variable focused on local growing seasons is beyond the scope of this paper.

WDI data are used to measure average aggregate investment and government consumption as a share of GDP. Non-agricultural value added is from the WDI and is measured in constant 2005 dollars to net out changes in relative prices. We blend it with FAOSTAT data on non-agricultural labor force to create a measure of non-agricultural value added per worker in constant dollars.

The sample includes only developing countries with available data, since the main drivers of growth in high-income economies are assumed to be innovation and increasing returns to scale in non-agricultural sectors. We use the middle of sample time period (1985) for country classification. The World Bank income ceiling for developing country status in 2012 was $12,615, and given that the WDI's GNI per capita data is reported in 2000 U.S. dollars, we deflate the ceiling to $9,699, and then keep only countries that had below that income in 1985 (keeping all post 1985-observations regardless of their income trajectory). The sample excludes small economies–defined as those with populations of less than 1 million in 1985–and developing economies in Europe, since their agricultural trajectories have been part of the process of temperate latitude technology transfer and were also affected by Soviet-era socialism. Socialist economies are excluded since their mechanism linking agriculture to structural change was likely skewed by central planning, particularly with regards to rural-to-urban migration and linkages otherwise working through price mechanisms in market economies. We exclude IMF-designated fuel exporters (Algeria, Angola, Congo, Iran, Libya, Nigeria, Oman, Trinidad and Tobago and Venezuela) and major diamond producers (Botswana, Guinea and Namibia).^10^ Unlike most developing countries these mineral exporters abound in foreign exchange to finance complementary inputs to labor in the non-agriculture sectors, and they are unlikely to have the low aggregate savings rates of countries where much of the population is in low-productivity farming, thus breaking some of the links between agricultural productivity and structural change mentioned earlier. This leaves 75 countries with data on cereal yields and fertilizer, although we limit the sample in the reduced form cereal yield specifications to 69 countries that have data on all variables. In the estimations for economic growth, labor share, and non-agriculture value added we opt for keeping a consistent number of countries that have data for all variables, thus forming a panel of 58 countries (note that some countries lack data during earlier years). The entire sample spans 1965–2000; however, the economic growth, labor share and NAVA estimations include lags which limit the sample period to 1975–2000. The 75-country sample and 58-country subsample are listed in Appendix Table A.3.

## 5 Results

### 5.1 Cereal yield production functions

Table 2 presents results for fixed-effect regressions with cereal yield per hectare as the dependent variable, covering five-year intervals over the period from 1965–2000. For each representative observation, yields, precipitation, fertilizer, irrigation, tractors and the labor-land ratio are averaged across three years (t−1, t, and t+1) in order to focus on structural shifts as opposed to year to year volatility. Column I presents pooled OLS with year dummies. The coefficient on fertilizer is 7.85 and strongly significant, implying that a 1 kg/ha increase in fertilizer is associated with higher yields of nearly 8 kg/ha. In the absence of country fixed effects, we expect this coefficient to be biased upward, since country-specific characteristics such as capital stock and governance are likely to be positively correlated with both fertilizer use and yields.

**Table 2:** Regressions on Cereal Yield.

	Dependent variable: Cereal yield, tons per hectare (at time t, in 5 year intervals)							
	Pooled OLS	Fixed effects estimator						
*Independent variables*	(I)	(II)	(III)	(IV)	(V)	(VI)	(VII)	(VIII)
Fertilizer per hectare [t/ha]	7.85^***^	4.54^***^	4.48^***^	3.40^**^	3.28^**^	3.11*	3.28^**^	3.14^*^
	(2.24)	(1.62)	(1.60)	(1.58)	(1.59)	(1.61)	(1.60)	(1.81)
								
ln (Precipitation [mm])			0.39^**^	0.39^**^	0.36^**^	0.38^**^	0.37^**^	0.36^**^
			(0.16)	(0.16)	(0.15)	(0.16)	(0.15)	(0.15)
								
Modern seeds (%)				0.010^***^	0.010^***^	0.010^***^	0.010^***^	0.010^***^
				(0.003)	(0.003)	(0.003)	(0.003)	(0.003)
								
ln(Agricultural Labor/Land Ratio)					−0.20	−0.23	−0.19	−0.19
					(0.21)	(0.21)	(0.21)	(0.23)
								
Irrigation (%)						0.007		
						(0.010)		
								
Years schooling							0.02	
							(0.06)	
								
Tractors per 100 sq km								3.29
								(10.64)
								
N	463	463	463	463	463	463	463	463
(Within) R-squared	0.50	0.62	0.63	0.66	0.66	0.67	0.66	0.66
Countries	69	69	69	69	69	69	69	69
Country Dummies	N	Y	Y	Y	Y	Y	Y	Y
Year Dummies	Y	Y	Y	Y	Y	Y	Y	Y

*Notes*: Standard errors in parentheses, clustered by country.

All variables except schooling and modern seeds are 3 year means measured at 5 year intervals. E.g., “1970” measures means over 1969, 1970 and 1971. The subsequent value averages over 1974, 1975 and 1976.

Constant terms, year dummies and country dummies not reported to save space. ^***^p<0.01. ^**^p<0.05. ^*^p<0.1.

Column II introduces country fixed effects and the fertilizer coefficient drops considerably to 4.54. Column III adds (the natural log of) precipitation. The fertilizer coefficient is nearly unchanged at 4.48 and precipitation is significant with a coefficient of 0.39. This coefficient implies that for a country like Uganda with average yields of 1.3 tons (1300 kg) per hectare and average precipitation of 1202 mm (near the 1201 mm sample average), a one standard deviation increase of 78 mm of precipitation would be associated with yield increases of 25 kg per hectare. Though statistically significant, this coefficient likely suffers from attenuation bias due to measurement error in the nationally-averaged precipitation variable. In an unreported regression, we run year-to-year yields on fertilizer and precipitation and find a consistent coefficient of 0.3 on the precipitation variable.

Column IV introduces another critical element of the green revolution package, modern variety seeds, which is significant at the 1 percent level and substantive in magnitude. This is a pure productivity effect. A marginal one percentage point increase in modern seed use is linked to an extra 10 kg per hectare yield, independent of fertilizer. The inclusion of the seed variable results in a slight decline in the fertilizer coefficient to 3.4, substantiating the point that fertilizer-seed packages have complementary effects in boosting yields.

To round out the production function with a measure of labor, column V adds the agricultural labor-land ratio. The variable is insignificant and has no perceptible effect on the other variables. It is worth noting that this table reports results for a consistent set of observations in all estimations, where the limiting variable in terms of data availability is for tractors. When column V is allowed to include all countries with available data (regression not shown), the larger sample results in a stronger association between labor-land ratio and yields, where the coefficient is −0.42 and significant at the 10% level. We opted for presenting a consistent sample across specifications to ease interpretation of coefficients.

Column VI introduces irrigation, the other main source of water for cereal crops. Column VII introduces human capital measured in average total years of schooling. Column VIII introduces the tractors variable to test for the effects of high-cost physical machinery. While these three variables have the expected positive sign, they are not statistically significant in the presence of country and time fixed effects.

#### 5.1.1 Instrumenting for fertilizer use

In order to gain better causal estimates for agricultural inputs on yields, we employ an instrumental variable framework in Table 3. The sample increases to 75 countries when not limited by the availability of irrigation, tractor and schooling variables as in the regressions of Table 2, though only 70 countries have data for precipitation, modern seeds, and our instrument.

**Table 3:** Regressions on Cereal Yield using IV.

	*Dependent variable*						
	Yields	Fertilizer	Yields	Fertilizer	Yields	Fertilizer	Yields
	(tons/ha)	(tons/ha)	(tons/ha)	(tons/ha)	(tons/ha)	(tons/ha)	(tons/ha)
	(I)	(II)	(III)	(IV)	(V)	(VI)	(VII)
*Independent variables*	FE	2SLS		2SLS		2SLS	
Global Fert Price/Cost-Adjusted Distance		−0.00075^***^		−0.00063^***^		−0.00093^***^	
to Nitrogen Production Site		(0.0001)		(0.00011)		(0.0001)	
Fertilizer per hectare (t)	2.93^**^		9.22^***^		8.55^***^		8.88^***^
	(1.15)		(1.51)		(1.88)		(1.44)
							
ln (Precipitation [mm]) (t)	0.31^*^			−0.001	0.30^*^		
	(0.17)			(0.018)	(0.16)		
							
Modern seeds (%)	0.012^***^			0.002^***^	0.0001		
	(0.002)			(0.0005)	(0.007)		
							
Global Oil Price/Cost-Adjusted Distance						0.0025^***^	−0.007
to Nitrogen Production Site						(0.0006)	(0.007)
ln(Exchange Rate)						−0.0016^*^	0.02^*^
						(0.0009)	(0.01)
							
Within R-squared	0.65	0.23	0.40	0.39	0.44	0.31	0.45
N	554	588		554		548	
Countries	70	75		70		73	
Kleibergen Paap F Test on First Stage		69.40		35.13		116.54	
Country Dummies	Y	Y	Y	Y	Y	Y	Y
Year Dummies	Y	Y	Y	Y	Y	Y	Y

*Notes*: Standard errors in parentheses, clustered by country in both first and second stages.

All variables except schooling and modern seeds are 3 year means measured at 5 year intervals.

E.g., “1970” measures means over 1969, 1970 and 1971. Constant terms, country dummies, time dummies not reported. ^***^p<0.01. ^**^p<0.05. ^*^p<0.1.

Column I repeats the country fixed effects regression from column IV in Table 2, using the larger sample. Column II then instruments fertilizer use with the fertilizer price-distance instrument in the first stage, resulting in a strongly significant coefficient and a first stage F-statistic of 69.40, above the usual threshold value of 10 for strong instruments (Staiger and Stock, 1997). The negative coefficient indicates that rises in global fertilizer price cause lower fertilizer use, in a pattern consistent with countries nearer fertilizer production sites experiencing larger proportional shocks. In column III, the second stage regression results in a coefficient for fertilizer of 9.22, suggesting that one kg/ha increase in fertilizer causes a 9 kg/ha increase in yield. Note that this is more than twice the magnitude of the fixed effects regressions of Table 2, suggesting that measurement error or omitted variable bias might have been significantly attenuating the estimates in the OLS regressions.

To provide a sense of magnitudes, the mean value of the instrument is 28.4 and the standard deviation is 30.7. One can consider a price shock of 10%, and compare a country with a cost-distance measure roughly one standard deviation above the mean (Mali, with a cost-distance of 9.60) with another country roughly one standard deviation below (Jamaica, with a cost-distance of 2.03). The coefficient and 10% price decrease imply that Mali would experience an 0.8 kg/ha increase in fertilizer use,^11^ while Jamaica would experience a 3.7 kg/ha increase. Given that Mali's fertilizer use in the sample averages 6.5 kg and ranges from 0.5 to 14 kg, while Jamaica averages 143 kg and ranges from 115 kg to 208 kg, the magnitude of the price effect seems plausible. Using the second stage coefficient of 9.22, a 10% fertilizer price decrease would increase yields by 7 kg/ha in the former versus 34 kg/ha in the latter. Columns IV and V repeat the instrumented first and second stages with controls for precipitation and modern seeds; the sample reduces from 75 to 70 countries, and both the coefficient on the instrument in the first stage and on fertilizer in the second stage remain strongly significant at the 1 percent level. Precipitation also has a positive coefficient in the second stage, though it is significant only to the 10 percent level.

Modern seeds are fertilizer responsive, and the variable is correlated to fertilizer use (as evidenced in the first stage), which is likely why the variable is insignificant in the second stage. Although diffusion of modern varieties within the sample period was notably driven by international public research bodies, the complementary nature of agronomic inputs suggests that rational farmers facing high fertilizer prices may choose to reduce both the use of fertilizer and other inputs. This makes it harder to distinguish the fertilizer effect on yield in isolation from other inputs. However, our primary aim is to study the effect of yield increases on structural change. As such, we can interpret our instrument as capturing exogenous variation in input use as a function of fertilizer prices. Regardless, the specifications in Table 3 provide confidence that the instrument for fertilizer use is valid and strong, and that fertilizer and its complementary inputs are important macro determinants of cereal yields. Countries facing greater barriers to fertilizer access will have a more difficult time boosting cereal yields.

Columns VI and VII test the robustness of the result to including two potential threats to the validity of the instrument. The first control divides the global price of crude oil by the same distance decay function we use to construct our instrument. This serves to assuage two potential concerns. The first possibility is that the distance to fertilizer production sites is proxying for access to global markets, and that our instrument is reflecting the differential effect of global business cycles on each country according to how far they are from the large global economies. Since the global oil price is highly correlated to the business cycles of large economies, finding an effect of our instrument on fertilizer consumption after adding this control suggests that fertilizer consumption is being affected by changes in global fertilizer prices independent of the fluctuations in oil prices and business cycles abroad. A second concern that this control addresses is the possibility that our instrument is capturing the effect of global changes in energy prices as opposed to the effect of fertilizer prices, especially since we have established the nitrogen fertilizer production has a cost advantage near natural gas deposits.

Finally, a different test for whether our instrument is proxying for connectedness to large economies is to consider that international price variation for commodities may differentially affect countries through their exchange rates to the dollar. We therefore control for the country's exchange rate. Even when adding these global oil price and exchange rate controls, the instrument continues to be highly significant and consistent in the first stage in column VI, despite there being an association between global oil price (decayed by distance) and fertilizer usage. The second stage coefficient on yields is 8.88, almost unchanged from the coefficient in column V.

### 5.2 Economic growth and labor share equations

#### 5.2.1 Growth in GDP per capita

As mentioned earlier, short-term increases in yield should appear directly in the GDP accounts, if land under cultivation is relatively fixed in the short term and agricultural output constitutes a sizable share of GDP. Table 4 presents fixed effects OLS estimators for Eq. (3), covering five-year growth periods from 1965 to 2000. Consistent with the growth literature (e.g., Caselli et al., 1996, Barro and Sala-i-Martin, 2004), the coefficient on lagged GDP per capita is close to 0.7, suggesting a convergence coefficient of approximately −0.06 per annum.

**Table 4:** Regressions on GDP per capita.

	Dependent variable							
	ln (GDP per capita)		Yield (t/ha) (t-1)	ln (GDP pc)	Yield (t-1)	ln (GDP pc)	Yield (t-1)	ln (GDP pc)
	(I)	(II)	(III)	(IV)	(V)	(VI)	(VII)	(VIII)
*Independent Variables*	FE	FE	2SLS		2SLS		2SLS	
Global Fert Price/Cost-Adj. Distance to			−0.008^***^		−0.008^***^		−0.009^***^	
Nitrogen Production Site (t-1)			(0.002)		(0.002)		(0.002)	
ln (GDP per capita) (t-5)	0.72^***^	0.72^***^	0.42^**^	0.65^***^	0.50^**^	0.59^***^	0.43^**^	0.58^***^
	(0.04)	(0.04)	(0.19)	(0.13)	(0.22)	(0.10)	(0.17)	(0.11)
								
Yield (tons/ha) (t-1)	0.08^***^	0.08^***^		0.37^***^		0.28^***^		0.37^***^
	(0.02)	(0.02)		(0.07)		(0.07)		(0.08)
								
Ave. Investment (t-5 to t-1)	0.009^***^	0.009^***^			−0.015^**^	0.012^***^		
	(0.001)	(0.002)			(0.007)	(0.002)		
								
ln (Inflation (t-5 to t-1))	−0.07^***^	−0.07^***^			0.003	−0.07^***^		
	(0.02)	(0.02)			(0.04)	(0.03)		
								
Gov′t Consumption as % of GDP (t-5 to t-1)	−0.007^***^	−0.009^***^			−0.003	−0.006		
	(0.002)	(0.002)			(0.01)	(0.004)		
								
Total Fertility Rate (t-5)	−0.036^*^	−0.031			−0.01	−0.03		
	(0.018)	(0.025)			(0.08)	(0.03)		
								
Global Oil Price/Cost-Adj. Distance to							−0.018^**^	0.006
Nitrogen Production Site (t-1)							(0.008)	(0.005)
ln (Exchange Rate) (t-1)							0.016	−0.02^***^
							(0.011)	(0.006)
								
ln(Global Food Price × Fitted Trade Share) (t-1)							−0.08^**^	0.05
							(0.04)	(0.04)
					
N	327	264	269		264		268	
Countries	64	58	58		58		57	
Within R-squared	0.87	0.83	0.58	0.61	0.61	0.77	0.57	0.65
Kleibergen Paap F Test on First Stage			18.62		12.73		28.95	
								
Country Dummies	Y	Y	Y	Y	Y	Y	Y	Y
Year Dummies	Y	Y	Y	Y	Y	Y	Y	Y

Standard errors in parentheses, clustered by country in both first and second stages.

All variables are 3 year means measured at 5 year intervals. E.g., “1970” measures means over 1969, 1970 and 1971. The subsequent value averages over 1974, 1975 and 1976.

Constant terms and country and time dummies not reported to save space. ^***^p<0.01. ^**^p<0.05. ^*^p<0.1.

Our main variable of interest is a lagged value of cereal yield, which has a very large and significant coefficient of 0.08 in column I. The within-country standard deviation of yields is approximately 500 kg/ha (0.5 tons/ha), so we proceed to interpret the instrumented yield coefficient in terms of a marginal increase of 0.5 tons. The coefficient implies that a half ton per hectare increase in yields is linked to a 4 percent increase in GDP per capita. The remaining variables are common in cross-country growth equations. Investment over the previous five years is positively correlated with growth, while inflation, government consumption as a percentage of GDP, and total fertility rates are all negatively correlated with growth. Note that column I does not limit the sample, while column II limits the sample to the 58 countries that have data on non-agricultural value added per worker. For comparability we retain the 58-country sample moving forward. Keeping this consistent sample throughout the analysis limits the time period to start in 1970, since the NAVA estimations involve longer lags in the independent variables. In unreported results, allowing a larger sample in Table 4 leads to consistent and significant coefficients on the yield variable.

We employ the instrumental variables framework to look at how shocks to yield through the fertilizer channel might show up in GDP, both contemporaneously and with a lag. Column III instruments for yields using the same instrument described above, and then GDP per capita is regressed in a second stage on the fitted value for yields in column IV. The first stage results in a highly significant coefficient on the instrument of −0.008, and the first-stage F-statistic on the instrument of 18.62 indicates that the instrument is adequately strong. The second stage coefficient on yield is significant at the 1 percent level and equal to 0.37, four times larger than the OLS regression of column I. The magnitude implies that a 0.5 ton increase in yield leads to a 20 percent higher GDP per capita.^12^

In columns V–VI, we control for the other elements of common growth regressions (investment, inflation, government consumption, and the total fertility rate). The first stage coefficient on the instrument continues to be significant and the F-statistic of 12.73 is above the strong instrument threshold, while the second-stage coefficient on yield is now 0.28. This implies that a half ton increase in cereal yields leads to a 15% higher GDP per capita, even when controlling for the 5-year lag of GDP. While this may seem like a surprisingly large result, one should keep in mind that in the 1960s, agriculture constituted over 30% of GDP in many countries. In countries where subsistence farming dominates and yields are around 1 ton per hectare, then a one-half ton increase in yields mechanically increases GDP by 15%. In fact, in an unreported result, when we limit the sample in regressions V–VI to the 30 countries above the median (29%) percentage of GDP in agriculture in 1960, the coefficient on yield in the second stage is 0.42. This is consistent with the theory that yield increases should boost GDP more in agriculture-dependent countries.

The increase in the coefficient from fixed effects to the 2SLS specification merits discussion. As mentioned earlier, one of several things could be at work. The first possibility is that OLS estimates suffer from large attenuation bias due to severe measurement error, which is prevalent in aggregate agricultural data in low-income countries (Jerven, 2010). A second possibility is that the larger 2SLS coefficient results from a violation of the exclusion restriction, such that the measured effect of yields on GDP is inappropriately capturing the effect of other omitted variables correlated to the instrument. For this reason, columns VII–VIII confirm that our results are robust to including controls for global oil price and exchange rates to the dollar. We also control for variation in global food prices interacted with the 1985 trade shares predicted by distance to trade partners from Frankel and Romer (1999). This tests whether the exclusion restriction might be violated through having fertilizer prices correlated to food prices, and whether the distance decay function in our instrument is confounding with connectedness to global markets. Finally, in an unreported result we interact the year dummies with the distance-based fitted trade shares from Frankel and Romer (1999) to allow global trends to vary by different countries' proximity to global markets. Our results remain unchanged.

**Fig. 8:**
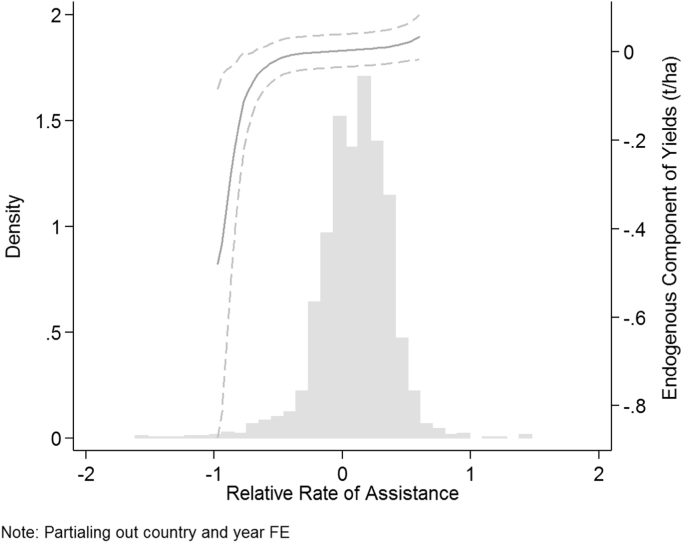
Yields and relative rates of assistance to agriculture.

A third explanation for why OLS estimates are attenuated is that an omitted variable correlated to high yields and low GDP per capita growth results in downward bias in OLS. Likely candidates for omitted variable bias include policies that distort agricultural markets through subsidies, trade barriers, or otherwise. We employ data from the World Bank's “Updated National and Global Estimates of Distortions to Agricultural Incentives, 1955 to 2011” from Anderson and Nelgen (2013) and discussed in detail in Anderson et al. (2013) in order to differentiate countries according to the degree to which they adopted policies distorting agriculture relative to the non-agricultural sector. They construct a “Relative Rate of Assistance” measure, which is the ratio between Nominal Rates of Assistance to agriculture and to non-agricultural sectors and therefore provides “an international comparable indication of the extent to which a country's sectoral policy regime has an anti- (or pro-) agricultural bias.” First, we explore the hypothesis that these kinds of policies could generate a downward bias in the OLS coefficient of yields on GDP by looking at the potentially endogenous portion of yields (using our IV) and testing whether high-distortion countries have systematically different yields after partialing out the exogenous variation in yields. These residual yields left after partialing out our instrument and the fixed effects are graphed against the RRA variable in Fig. 8. The graph shows a positive relationship between RRA and the endogenous component of yields. In particular, countries with large distortionary anti-agriculture policies (RRA less than −0.5), have significantly lower values for the endogenous component of yields. Given that the strongest effects on yield in the figure occur at RRA levels below −0.5, we define “high distortion” country-years as those where the RRA was less than −0.5. If these policies are an omitted variable generating a negative correlation between national income growth and yields, then the countries with high levels of distortion should have an attenuated relationship between yields and income. We test this in regression (I) of Table 5, and find that it is indeed the case. The interaction between yields and the high distortion variation is −0.061, indicating that countries with large agriculture price distortions relative to other sectors have a smaller coefficient of yields on GDP in the OLS regression. This suggests that these distortionary policies generate a downward omitted variable bias in the FE regressions in the paper, and are a reason for why the IV estimates produce a larger coefficient.

**Table 5:** Anti-Agriculture Policies and Extensification in GDP per capita Regressions.

	Dependent variable	
	ln (GDP per capita)	
	(I)	(II)
*Independent Variables*	FE	FE
ln (GDP per capita) (t-5)	0.72^***^	0.72^***^
	(0.04)	(0.05)
		
Yield (tons/ha) (t-1)	0.08^***^	0.09^***^
	(0.02)	(0.02)
		
High Anti-Agriculture Distortion (RRA < -0.5)	0.08	
	(0.08)	
		
Yield (tons/ha) (t-1) * High Distortion	−0.061^*^	
	(0.034)	
		
Yield (tons/ha) (t-1) * High Extensification		−0.040^*^
		(0.022)
		
Ave. Investment (t-5 to t-1)	0.009^***^	0.009^***^
	(0.002)	(0.002)
		
ln (Inflation (t-5 to t-1))	−0.07^***^	−0.07^***^
	(0.02)	(0.02)
		
Gov′t Consumption as % of GDP (t-5 to t-1)	−0.008^***^	−0.009^***^
	(0.002)	(0.002)
		
Total Fertility Rate (t-5)	−0.033	−0.030
	(0.025)	(0.025)
		
N	264	264
Countries	58	58
Within R-squared	0.84	0.84
		
Country Dummies	Y	Y
Year Dummies	Y	Y

Standard errors in parentheses, clustered by country.

All variables are 3 year means measured at 5 year intervals. E.g., “1970” measures means over 1969, 1970 and 1971.

Constant terms and country and time dummies not reported to save space. ^***^p<0.01. ^*^p<0.1.

A second reason for why the average yields per hectare may be negatively correlated to GDP growth is if farmers are expanding production along the extensive margin. This may be especially likely in countries where farmers face barriers to using more agronomic inputs due to credit constraints or risk aversion. If the cropland expansion were into marginal or otherwise lower quality lands, then this would translate to lower average yields per hectare even as total production increases. Since our empirical exercise is to estimate the causal effect of increased input use on GDP growth and structure change, this effect of decreasing average yields with extensification confounds our estimate in the OLS. We collect data from the FAO on total land area harvested by crop, and look at the correlation of cereal yields per hectare and the log of total land area harvested under cereals. The correlation after partialing out country and year fixed effects is −0.19, supporting the hypothesis that extensification can lower average yields per hectare even as total production increases. In regression II of Table 5, we define high extensification countries as those that had over 75% growth in the total cereals area harvested between 1960–2000.^13^ The high extensification countries have a lower estimated coefficient of yields on GDP (coefficient is −0.04), once again consistent with the hypothesis that OLS could be biased downwards if farmers respond to income growth by expanding production along the extensive margin. Since our IV is measuring the effects of agricultural intensification, it stands to reason that the IV estimate will be larger than OLS.

It is important to consider the implications of specifying yields in levels. We show in the robustness section that specifying yields in logs or using the log of agricultural value added per worker instead leads to results that are consistent but only marginally statistically significant. This is due to a weaker first-stage relationship when using yields in logs, suggesting that variation in fertilizer price affects yields in low productivity countries proportionally more than in high productivity countries. This is plausible, given that farmers in low-productivity countries are likely poorer and therefore more sensitive to the price of inputs. The second stage results when using yields in levels suggests that economies with higher levels of agricultural productivity benefit equally from a one-ton increase in cereal yields as countries at lower levels of productivity. This is unlikely the case as countries enter high-income status, but we remind the reader that high-income countries are excluded from the sample, and many middle-income countries in the sample (such as Brazil and Argentina) have economies in which agriculture plays an important role.

**Fig. 9:**
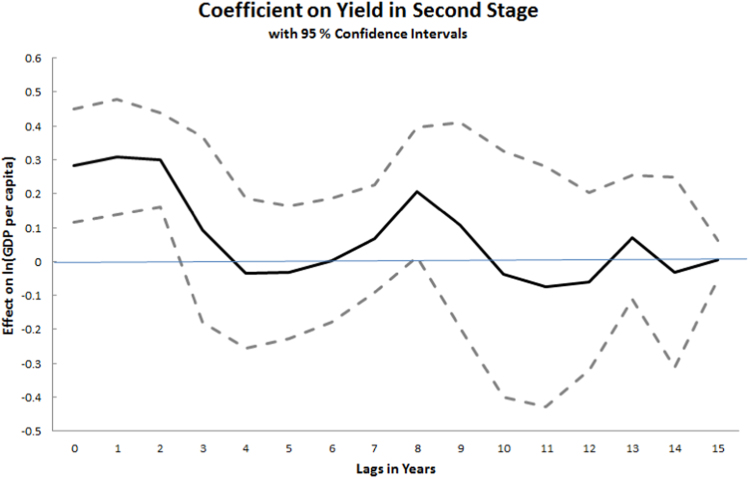
Coefficients on different lags of instrumented cereal yield in a specification following Table 4 Columns III–IV.

Table 4 uses a 1-year lagged value of yield, keeping in mind that both the GDP and yield variables are three-year moving averages. We tested from zero- to fifteen-year lags in the specification of columns III–IV in order to explore the lag structure of this causally identified effect of yield shocks on GDP, and found an effect in the contemporaneous year as well as one and two years later. The lagged coefficients with 95% confidence intervals are graphed in Fig. 9, and suggest a statistical relationship between a three-year moving average of GDP per capita centered at time t with yield at t, t−1 and t−2. We therefore opt to present our estimates using yield centered at t−1.

#### 5.2.2 Labor share in agriculture

**Fig. 10:**
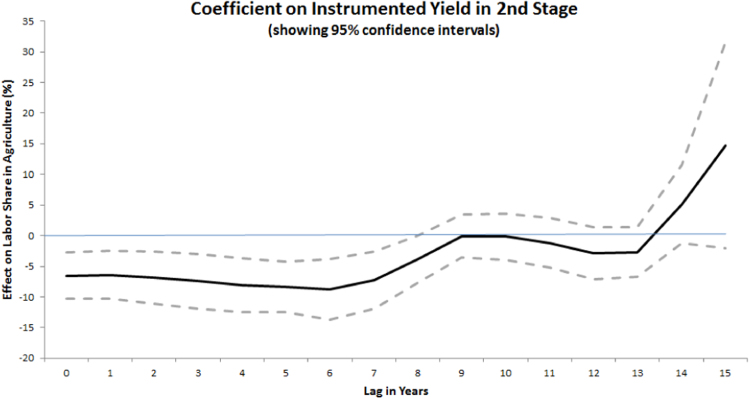
Coefficient on different lags of instrumented cereal yield in a specification following Table 6 Columns VII–VIII.

Since one would expect yield increases to raise GDP mechanically, a more appropriate measure of looking at whether yield increases are triggering structural change is to focus on labor movement out of agriculture. In Table 6 we test labor share in agriculture as the dependent variable. The mean share in the sample is 52 percent. Columns I–IV use OLS with country and year fixed effects, while V–XI instrument for yields. We first examine the lag structure, as shown in Fig. 10. Note that higher (instrumented) yields are correlated with lower labor shares in agriculture both contemporaneously and during the next seven years before the effect dissipates. We use a 5-year lag on yields in the Table 6 estimations.

**Table 6:** Regressions on Labor Share in Agriculture.

	Dependent variable										
	Labor Share in Agriculture				Yield (t/ha) (t-5)	L Share in Ag.	Yield (t-5)	L Share in Ag.	Yield (t-5)	L Share in Ag.	L Share in Ag.
	(I)	(II)	(III)	(IV)	(V)	(VI)	(VII)	(VIII)	(IX)	(X)	(XI)
*Independent Variables*	FE	FE	FE	FE	2SLS		2SLS		2SLS		2SLS
Global Fert Price/Cost-Adj. Dist. to					−0.005^***^		−0.006^***^		−0.005^***^		
Nitrogen Production Site (t-5)					(0.001)		(0.001)		(0.001)		
Yield (t-5)	−3.30^**^	−3.29^**^	−1.98	−3.98^**^		−9.43^***^		−9.13^***^		−11.28^***^	−10.72^***^
	(1.59)	(1.60)	(1.52)	(1.55)		(1.82)		(1.82)		(1.80)	(2.07)
											
Ave. Investment (t-5 to t-1)		−0.02	−0.04				0.002	−0.04			
		(0.07)	(0.06)				(0.005)	(0.08)			
											
ln (Inflation (t-5 to t-1))		0.13	0.06				−0.05	−0.36			
		(0.19)	(0.45)				(0.04)	(0.57)			
											
Gov't Consumption, % of GDP (t-5 to t-1)			0.23^**^				−0.007	0.17			
			(0.10)				(0.011)	(0.12)			
											
Total Fertility Rate (t-5)			2.50^***^				−0.13^*^	1.56			
			(0.69)				(0.07)	(0.98)			
											
Yield (t-5)^*^ Cereal Exporter				6.12^***^							9.78^***^
				(1.99)							(3.16)
											
Global Oil Price/Cost-Adj. Dist. to									0.0001	−0.13^***^	
Nitrogen Production Site (t-5)									(0.006)	(0.04)	
ln (Exchange Rate) (t-5)									0.003	0.24	
									(0.01)	(0.17)	
											
ln(Global Food Price^*^ Fitted Trade Share) (t-5)									−1.26^***^	7.64^***^	
									(0.02)	(1.19)	
								
N	269	269	264	269	269		264		268		269
Countries	58	58	58	58	58		58		57		58
Within R-squared	0.78	0.78	0.83	0.79	0.52	0.70	0.57	0.74	0.52	0.66	0.72
Kleibergen Paap F Test on First Stage					39.95		20.52		18.37		
											
Country Dummies	Y	Y	Y	Y	Y	Y	Y	Y	Y	Y	Y
Year Dummies	Y	Y	Y	Y	Y	Y	Y	Y	Y	Y	Y

Standard errors in parentheses, clustered by country in both first and second stages.

All variables are 3 year means measured at 5 year intervals. E.g., “1970” measures means over 1969, 1970 and 1971. The subsequent value averages over 1974, 1975 and 1976. Columns V, VII, and IX are first-stage regressions for VI, VIII and X, respectively. First stage for column XI omitted to save space.

Constant terms and country and time dummies not reported to save space. ^***^p<0.01. ^**^p<0.05. ^*^p<0.1.

Column I indicates the strong association between labor share in agriculture and lagged yield, even when controlling for country and year fixed effects. The coefficient of −3.3 indicates that a 0.5 ton increase in yields is associated with a 1.65 percentage point lower share of the labor force in agriculture 5 years later. Column II adds investment and inflation as controls. Neither is significant, and the coefficient on lagged yield does not change. Column III adds government consumption and the fertility rate. Government consumption is positively correlated with labor share in agriculture, which might be an indication of excessive government intervention in the economy delaying structural change. Higher fertility is also associated with a higher labor share in agriculture in subsequent years. In general, higher fertility increases demand for food and thus for agricultural labor, while in the reverse causal direction agrarian societies tend to have higher fertility rates due to high mortality, low returns to education and demand for labor on the farm. Note that the coefficient on lagged yield is smaller and not significant, suggesting that, in the absence of an identification strategy, several of the independent variables might be trending together, and the correlation between them is attenuating the association between yields and labor share.

Column IV explores whether the effect of yield is differentiated across countries as Matsuyama (1992) predicts: increased agricultural productivity in open economies may lead to specialization in agriculture and countries pursuing that comparative advantage, thus pulling labor into agriculture. We use FAO data to divide each country's cereal exports by total production, and find that in our sample the median country exports 0.4% of production (this suggests that Matsuyama's predictions of how economies exporting staple foods would respond to productivity increases does not apply to most of the developing world). We designate as cereal exporters those countries that exported at least 10% of their cereal production over at least half of the time period in our study. These are Argentina, Jordan, South Africa, Thailand, Uruguay and Zimbabwe. Columns IV then interacts the yield variable with the cereal exporter indicator variable; the resulting coefficient on yield is −3.98, while the coefficient on the interaction is 6.12.^14^ Countries that are cereal exporters, therefore, display a different dynamic from the rest: increased yields lead to (small) increases in the labor share in agriculture, as Matsuyama (1992) would predict. We return to the implications of this finding for public policy in the conclusion.

Columns V and VI instrument for yields, again using the fertilizer price-distance variable. The instrument continues to be strongly correlated with yields, the first stage F-statistic on the instrument is 39.95, and the second stage coefficient on yield increases to −9.43, significant at 1 percent level. This suggests that a 0.5 ton increase in yields causes the labor share in agriculture to decrease by nearly 5 percentage points in the next five years. The result is consistent when controlling for investment, fertility rate, inflation and government consumption in VII–VIII. As above, columns IX–X check for potential violations of the exclusion restriction by controlling for the global oil price interacted with the inverse distance we use in our instrument, as well as controlling for the country's exchange rate. We also control for the global food price interacted with distance-based fitted 1985 trade shares from Frankel and Romer (1999). The results are robust to including these controls (in fact, the coefficient rises to -11.28). As with our analysis on GDP, in an unreported regression we interact the year dummies with the fitted trade shares to allow for differential global trends according to countries' proximity to global markets, and results are unchanged. Finally, column XI shows the second stage from interacting the yields variable (and the instrument) with the cereal exporters binary variable as defined for column IV. The resulting coefficient on the interaction is positive and significant, and the magnitude suggests that the impact of yields on labor shares in most countries is absent in the case of cereal exporters.

As with the results on GDP, the higher coefficient on yields in the 2SLS framework suggests that OLS is biased downwards due to attenuation from measurement error, or due to an omitted variable that is negatively correlated to yields and to slower labor movement out of agriculture (such as distortionary policies or extensification by farmers, as discussed earlier). Given that the results on labor share are our preferred evidence for the causal effects of yield increases on structural transformation, we employ a test developed by Conley et al. (2012) to explore the possibility of an exclusion restriction violation. The test is discussed in the Appendix, and suggests that results in Table 6 are robust to a small relaxation of the exclusion restriction. Our results on GDP are even more robust: the test suggests that negating our results would require that most of the effect of fertilizer price changes on income occur through a channel other than yields, which is highly unlikely.

#### 5.2.3 Growth in non-agricultural value added

Arguably the most ambitious test for links between agricultural productivity and structural change is to examine the links to economic activity entirely outside of agriculture. Table 7 does this by testing non-agricultural value added per non-agricultural worker (NAVA) as the dependent variable. Since we expect a delay between having a boost in yields and spillovers to the non-agriculture sector, and there is no theoretical prior on the timing, we first explore the lag structure.

**Table 7:** Regressions on Non-agricultural Value Added per Worker.

	Dependent variable										
	ln(NAVA)	ln(NAVA)	ln(NAVA)	Yield (t-9)	ln(NAVA)	Yield (t-9)	ln(NAVA)	Yield (t-9)	ln(NAVA)	Yield (t-9)	ln(NAVA)
	(I)	(II)	(III)	(IV)	(V)	(VI)	(VII)	(VIII)	(IX)	(X)	(XI)
*Independent variables*	FE	FE	FE	2SLS		2SLS		2SLS		2SLS	
9-year lag in Global Fert Price/Cost-				−0.008^***^		−0.008^***^		−0.007^***^		−0.010^***^	
Adj. Dist. to Nitrogen Production Site				(0.001)		(0.001)		(0.001)		(0.003)	
5-year lag ln(non ag value per worker)	0.88^***^	0.73^***^	0.77^***^	0.42^**^	0.79^***^	0.50^***^	0.63^***^	0.52^***^	0.69^***^	0.36^**^	0.82^***^
	(0.05)	(0.07)	(0.06)	(0.16)	(0.08)	(0.16)	(0.08)	(0.17)	(0.08)	(0.14)	(0.08)
											
9-year lag yield	0.05^*^	0.06^*^	0.03		0.23^*^		0.24^**^		0.17^**^		0.17
	(0.03)	(0.03)	(0.03)		(0.13)		(0.10)		(0.08)		(0.13)
											
Ave. Investment (t-5 to t-1)		0.01^***^	0.01^***^			−0.010^**^	0.01^***^	−0.010^**^	0.01^***^		
		(0.003)	(0.003)			(0.005)	(0.003)	(0.005)	(0.003)		
											
ln(Inflation (t-5 to t-1))		−0.10^**^	−0.10^**^			0.001	−0.10^**^	0.007	−0.10^**^		
		(0.05)	(0.04)			(0.03)	(0.05)	(0.03)	(0.05)		
											
Gov't Consumption as % of GDP			−0.005					−0.006	−0.004		
(t-5 to t-1)			(0.003)					(0.007)	(0.004)		
Total Fertility Rate (t-5)			−0.04					−0.13^*^	−0.02		
			(0.03)					(0.08)	(0.04)		
											
9-year lag in Global Oil Price/Cost-										0.01	−0.002
Adj. Dist. to Nitrogen Production Site										(0.01)	(0.002)
9-year lag in ln(Exchange Rate)										0.015^**^	−0.001
										(0.006)	(0.006)
							
N	335	264	260	264		264		260		262	
Countries	65	58	58	58		58		58		57	
Within R-squared	0.77	0.79	0.80	0.60	0.68	0.61	0.75	0.64	0.78	0.61	0.69
Kleibergen Paap F Test on First Stage				34.25		30.59		26.96		11.50	
											
Country Dummies	Y	Y	Y	Y	Y	Y	Y	Y	Y	Y	Y
Year Dummies	Y	Y	Y	Y	Y	Y	Y	Y	Y	Y	Y

*Notes*: Standard errors in parentheses, clustered by country in both first and second stages.

NAVA: Non-agricultural value added per worker

All variables are 3 year means measured at 5 year intervals. E.g., “1970” measures means over 1969, 1970 and 1971. The subsequent value averages over 1974, 1975 and 1976.

Constant terms, year dummies, and country dummies not reported to save space. ^*^p<0.1. ^**^p<0.05. ^***^p<0.05.

**Fig. 11:**
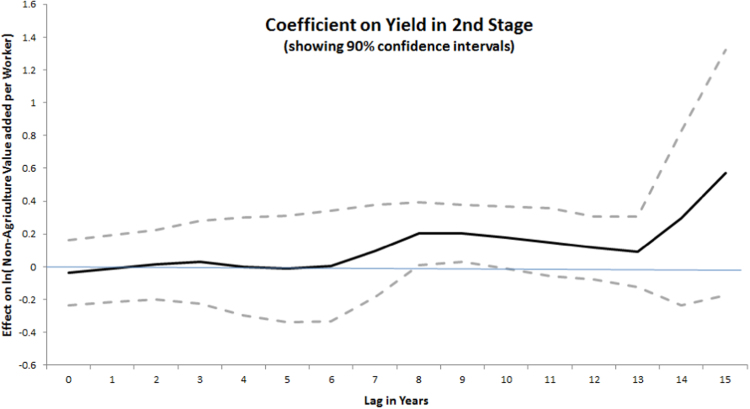
Coefficients on different lags of instrumented cereal yield in a specification following Table 7 Columns VI–VII.

Fig. 11 shows the results of regressions of non-agricultural value added per worker tested against 15 respective lags (from t to t−15) of instrumented cereal yields. Two things are evident when comparing this graph to the one relating cereal yields and GDP per capita. First, the statistical signal is weaker (note that we are using 90% confidence intervals in this graph), although the effect remains positive at every lag. However, we note that the statistical significance is only evident in two lags, which cannot be distinguished from the expected false positive rate at 90% confidence when looking at 15 lags. Therefore, the results on NAVA should be interpreted as only suggestive. The second observation is that the impact of yields on the non-agricultural sector productivity appears to occur with a longer lag (about 8–10 years). This longer delay might indicate that the relationship between yields and non-agricultural value added per worker might occur through slower-moving channels such as movement of labor from agriculture to non-agriculture, as opposed to faster channels like relative price changes or increases in food production immediately generating disposable income for investment in other sectors.

Given the lag structure evidence, Table 7 shows results for non-agricultural value added regressions using a 9-year lag on cereal yield.^15^ Column I presents the fixed effects regression with no controls. The 9-year lagged cereal yields are positively associated with increases in non-agricultural worker productivity although only significant at the 10 percent level. The coefficient implies that 0.5 ton per hectare yield increases are associated with a 2.5 percent higher non-agriculture productivity level around 9 years later. Column II adds investment and inflation; the lag NAVA coefficient drops from 0.88 to 0.73, similar to the coefficient in the GDP regression, while the yield coefficient is 0.06 and falls just short of 5 percent significance. Investment rates are positively correlated with non-agricultural productivity growth, while inflation is negatively correlated. Column III adds government consumption and the total fertility rate. The coefficient on yield remains generally consistent at 0.03, although it is not significant in this specification. Neither government consumption nor the total fertility rate are significant.

The rest of Table 7 employs the same identification strategy as in the GDP per capita regressions by using our fertilizer price-distance instrument for yields. The two-staged least squares results in columns IV–V employ no macroeconomic controls; the instrument is highly significant in the first stage, and the F-statistic of 34.25 indicates that the instrument is strong. In the second stage, the coefficient on the instrumented lagged cereal yield is significant and rises in magnitude to 0.23, significant to 10% levels. This suggests that an exogenous 0.5-ton increase in cereal yields leads to a 12 percent higher non-agricultural productivity nine years later, which translates to a 1.3 percent higher growth rate of annual productivity per worker. As was the case for results on labor shares, the specifications instrumenting for yields produce significantly higher coefficients on yield in the second stage, suggesting either attenuation bias from measurement error in the OLS specifications or an omitted variable that is increasing yields and decreasing growth in non-agricultural labor productivity (or vice versa).

Regressions VI and VII add investment and inflation over the previous five years as controls. The results are consistent: the instrument is significant and has an F-statistic of 30.59, and in the second stage the coefficient on the instrumented cereal yields is consistent and now significant at 5% levels. Investment and inflation are significant and have the expected signs. Regressions VIII and IX add government consumption and the lagged total fertility rate. The first stage results are effectively unchanged, with the instrument still highly significant and an F-statistic of 26.96. The second stage coefficient on cereal yields drops slightly to 0.17, suggesting a 0.5 ton boost in cereal yields leads to a 0.9 percent higher annual growth rate in non-agricultural productivity. Columns X and XI add the oil price and exchange rate controls as potential violations of the exclusion restriction, as discussed in previous tables. The resulting second-stage coefficient on yields is consistent at 0.17, however it loses statistical significance. Overall, Table 7 provides consistent IV results suggesting that exogenous half ton increases in yields lead to approximately 11 percent higher non-agricultural value added per worker a decade later, though we remain cautious about this evidence given the relatively weak statistical signal in Fig. 11. Nevertheless, this lends empirical support for the potential role of agriculture in promoting structural change.

Given that our instrument is based on exogenous variation in supplier access to fertilizer production sites, it is instructive again to compare the implications for Mali and Jamaica. In the former, a 10% negative price shock to global fertilizer prices would have the same fertilizer and yield effects as described earlier, and would increase GDP per capita by 0.3%, decrease the labor share in agriculture by 0.1 percentage points over the next five years, and increase labor productivity in the non-agricultural sector by 0.2% over the next nine years. Meanwhile, Jamaica would experience a GDP per capita increase of approximately 1.3%, a decrease in labor share in agriculture by 0.3 percentage points over the next five years, and an increase in non-agricultural labor productivity of around 0.8% over the next nine years.

### 5.3 Robustness checks

A potential concern with the instrument is the endogenous location of fertilizer production sites. We note that most of the sites are in developed countries, and thus not in our sample. The exceptions are the sites located in Argentina, Brazil, China, Egypt, India, Libya and Trinidad & Tobago. If fertilizer plants located endogenously as a result of improved economic outcomes, and if this nearby location triggered agricultural yield increases, then the instrument would be inappropriately coding for the endogenous location dynamics. To assuage this concern, we ran all regressions while dropping these seven countries (around 7–10% of the data depending on the regression). The results from regressions on labor shares remain qualitatively unchanged (the second stage coefficient on yields remains consistent and significant). In the case of non-agricultural value added, the second stage coefficient on yields remains consistent in magnitude, but loses statistical significance. The fact that the coefficients are consistent without exception suggests that the loss of significance is likely due to sample size issues and the results are not being driven by these seven countries. The second point to note regarding endogenous location of fertilizer production is that the Haber-Bosch process for nitrogen fertilizer production requires natural gas, which creates cost advantages to being close to a natural gas field. As mentioned earlier, the correlation between the distance from agricultural centroid to nearest fertilizer production site and the distance from agricultural centroid to nearest natural gas field is 0.76 for the countries in our sample.^16^

Given that we are presenting results in levels, we conduct an outlier analysis to make sure that our results are not being driven by a small number of observations. For both labor shares and non-agricultural value added per worker, we estimate the endogenous equations (yields without the instrument), calculate a Cook's distance, and make sure that our 2SLS results are all robust to excluding the observations with a Cook's distance above 1. Note that if we calculate Cook's distance on the first stage regression using the instrument, no observations are significant outliers. Only two observations in the endogenous labor share regression had a Cook's distance above 1 in the second stage (Benin and Cambodia in the year 2000). Excluding them from the 2SLS specification leads to no change in the point estimates in either the first or second stage. Similarly, when looking at regressions on NAVA per worker, three observations have Cook's distance above 1 in the second stage (Benin, Cambodia and Mongolia in 2000). Dropping these observations from 2SLS leaves the point estimates unchanged.

For completeness, we also examine the GDP and labor share regressions using yields in logs instead of levels, and present the results in Table 8. Jordan has large residuals in the relationship between log yields and the dependent variables, so we present regressions both including and excluding Jordan. All first-stage regressions have F-statistics on the instrument below 10, suggesting that the instrument is weak in these log specifications.^17^ In the GDP regressions, log yields have a positive coefficient on the second stage, but only weakly significant (when Jordan is excluded). The magnitude of 3.34 indicates that increasing yields by 0.5 tons/ha (31% above the sample mean yield of 1.6 tons/ha) would increase GDP per capita by 104%. This effect is larger than when analyzing yields in levels. In the regressions on labor shares, the instrument in the first stage is significant to the 10% level and the F-statistic is 3.56. The second stage coefficient is −37.71, suggesting that increasing yields by 0.5 tons/ha would decrease the labor share by 11.7 percentage points. Excluding Jordan in regressions VII and VIII leads to significance at 5% levels in both the first and second stages. Since the main regressions with yields in levels produce more conservative and more precise estimates, we opt for the levels regressions (with appropriate outlier tests) as the preferred specifications. As mentioned earlier, it is likely that the weaker first stage relationship when using yields in logs results from farmers in low productivity settings being more sensitive to input prices, such that a change in fertilizer prices does not lead to the same proportional change in yields across high- and low-productivity settings.

**Table 8:** Regressions with yields in logs.

	Dependent variable							
	ln (Yield) (t−1)	ln (GDP pc)	ln (Yield) (t−1)	ln (GDP pc)	ln (Yield) (t−5)	Labor Share in Agr.	ln (Yield) (t−5)	Labor Share in Agr.
	(I)	(II)	(III)	(IV)	(V)	(VI)	(VII)	(VIII)
*Independent Variables*	2SLS		2SLS		2SLS		2SLS	
Global Fert Price/Cost-Adj. Dist. to	−0.0012		−0.0009					
Nitrogen Production Site (t-1)	(0.0008)		(0.0006)					
ln (Yield) (t-1)		2.48		3.34^*^				
		(1.57)		(1.88)				
								
ln (GDP per capita) (t-5)	0.07	0.64^***^	0.07	0.57^*^				
	(0.07)	(0.24)	(0.06)	(0.31)				
								
Global Fert Price/Cost-Adj. Dist. to					−0.0013^*^		−0.0011^**^	
Nitrogen Production Site (t-5)					(0.0007)		(0.0005)	
ln (Yield) (t-5)						-37.71^*^		−50.24^**^
						(21.12)		(21.54)
				
N	269		264		269		264	
Countries	58		57		58		57	
Within R-squared	0.48	0.58	0.50	0.51	0.54	0.19	0.55	0.77
F Test on First Stage	2.04		2.46		3.56		4.51	
								
CountryDummies	Y	Y	Y	Y	Y	Y	Y	Y
Year Dummies	Y	Y	Y	Y	Y	Y	Y	Y

Standard errors in parentheses, clustered by country in both first and second stages.

All variables are 3 year means measured at 5 year intervals. E.g., “1970” measures means over 1969, 1970 and 1971. The subsequent value averages over 1974, 1975 and 1976.

Constant terms and country and time dummies not reported to save space. Regressions III/IV and VII/VIII exclude Jordan. ^*^p<0.1. ^***^p<0.01. ^**^p<0.05.

We also conduct the following tests: adding region- or country-specific linear trends to the regressions, considering agricultural value added instead of yields, and running the dynamic panel regressions using GMM instrumentation. Secular trends in our yield, labor share, and labor productivity variables as economic development proceeds might result in spurious correlations, or introduce omitted variable bias to our specifications given that development involves many economic characteristics changing together. To that end, Table 9 adds region-specific and country-specific linear trends to the IV regressions on labor shares and non-agricultural value added using geographic regions as defined by the World Bank. Columns I-II add regional linear trends to the labor share specification. The F-statistic on the instrument is 12.44, suggesting that the instrument is strong despite partialing out regional linear trends alongside country and year fixed effects. The second stage shows that the coefficient on instrumented lagged cereal yields remains significant and consistent in magnitude to our core results. Regressions III and IV employ country-level linear time trends instead of region-level trends, which significantly decreases the degrees of freedom in the model. The instrument is significant only to the 10% level in the first stage, and has an F-statistic of 3.16. The weak first stage leads to an insignificant coefficient on yields in the second stage, although the magnitude is larger than that of our core specifications and the sign continues to be negative. Regressions V–VI and VII–VII look at NAVA per worker, using region- and country-level trends, respectively. As with labor shares, the first stage regression is robust to including region-level linear trends, with the coefficient on the instrument remaining consistent, strongly significant, and the first-stage F-test having a value of 21.81. The second stage coefficient on yield is significant to the 10% level and consistent in magnitude with the results in Table 7 (0.29 compared to 0.23). Adding country-level linear trends significantly reduces the strength of the first stage (F-statistic decreases to 3.37), consequently precision is lost in the second stage estimate. Nevertheless, the coefficient remains consistent at 0.28. Our core results are robust to including regional linear trends. Adding country-level trends is the strongest statistical test, and although estimates become imprecise, we interpret the consistency of the results as supporting the overall findings.

**Table 9:** Adding region and country linear trends.

	Dependent variable							
		Labor Share in Agriculture		Labor Share in Agriculture		ln (non agriculture value		ln (non agriculture value
	Yield (t−5)		Yield (t−5)		Yield (t−9)	added per worker)	Yield (t−9)	added per worker)
	(I)	(II)	(III)	(IV)	(V)	(VI)	(VII)	(VIII)
*Independent variables*	2SLS		2SLS		2SLS		2SLS	
5-year lag in Global Fert Price/Cost-j	−0.004^***^		−0.0013^*^					
Adj. Dist. to Nitrogen Production Site	(0.001)		(0.0007)					
5-year lag yield		−8.52^**^		−16.12				
		(3.53)		(9.97)				
								
9-year lag in Global Fert Price/Cost-					−0.006^***^		−0.0030^*^	
Adj. Dist. to Nitrogen Production Site					(0.0014)		(0.0017)	
9-year lag ln(non ag value per worker)					0.26^**^	0.73^***^	−0.15	0.39^**^
					(0.10)	(0.10)	(0.17)	(0.16)
								
9-year lag yield						0.29^*^		0.28
						(0.15)		(0.47)
				
N	269		269		264		264	
Countries	58		58		58		58	
Kleibergen Paap F Test on First Stage	12.44		3.16		21.81		3.37	
								
Country Dummies	Y	Y	Y	Y	Y	Y	Y	Y
Year Dummies	Y	Y	Y	Y	Y	Y	Y	Y
Linear Trends	Region	Region	Country	Country	Region	Region	Country	Country

*Notes*: Standard errors in parentheses, clustered by country in both first and second stages.

All variables are 3 year means measured at 5 year intervals. E.g., “1970” measures means over 1969, 1970 and 1971. The subsequent value averages over 1974, 1975 and 1976.

Constant terms, year dummies, and country dummies not reported to save space. Regions include East Asia & Pacific, Latin America & Caribbean, Middle East & North Africa, South Asia, and sub-Saharan Africa. ^*^p<0.1. ^***^p<0.01. ^**^p<0.05.

While our analysis has studied the impact of agronomic yield increases on structural transformation, an alternative measure is to focus on labor productivity in agriculture. In the Appendix we consider agricultural value added per worker in agriculture, with the caveat that this represents labor productivity across the entire sector (including livestock and cash crops), and thus a measure less relevant for studying the impact of raising productivity in the staple food sector. In any case, the results (presented in Appendix Table A.4) remain consistent in the case of fertilizer raising agricultural labor productivity, and in turn raising GDP and reducing labor shares in agriculture. Finally, Appendix Table A.5 presents a NAVA growth framework using GMM instrumentation and finds similar agricultural productivity effects on value added in non-agricultural sectors.

## 6 Discussion and conclusion

Our analysis documents the strong links between agronomic inputs – fertilizer, water and modern seeds – and cereal yields per hectare, even after a variety of controls are introduced. We employ a combination of fixed effect and instrumental variable specifications to posit a causal economy-wide link between, first, input use and yields, and second, yields and various measures of economic growth and structural change. We construct a novel instrument exploiting the economic geography of fertilizer production, which together with global fertilizer price fluctuations allow for a statistically causal framework. The cross-country substantiation of both agricultural yield production functions and their links to various dimensions of economic growth and structural change are empirically consequential. Taking the coefficients from Table 3, a representative country with yields of 1500 kg/ha (1.5 t/ha) that introduces an input package to jump from, say, 15 kg/ha to 65 kg/ha of fertilizer use would be expected to see an average yield jump of 147–462 kg/ha (0.147–0.462 tons/ha); while increasing from 10 to 50 percent use of modern seed would be expected to increase yields by up to 480 kg/ha (0.48 tons/ha).

With regards to economic growth and structural change, the IV results suggest that boosting yields from 1,500 to 2,000 kg/ha (1.5 t/ha to 2.0 t/ha) is linked to a range of 14 to 19 percent increase in income per capita, and a 4.6 to 5.6 percentage point lower share of labor in agriculture five years later. There is also suggestive evidence that this yield boost is associated with approximately 9 to 12 percent higher non-agricultural labor productivity after roughly one decade. The estimated effects are identified based on exogenous variation in fertilizer prices, and are robust to the inclusion of controls for investment and standard macroeconomic policy indicator variables. The results suggest that land productivity promotes growth both by supporting changing labor shares and by increasing total factor productivity. Regressions focused on marginal effects of individual variables are not intended to evaluate nonlinear outcomes guided by Leontief-style agricultural production functions and discontinuous policy functions, so the results might underestimate the potential effects of yields.

The evidence in this paper points to strong potential yield and growth effects resulting from policy efforts to support adoption of a green revolution-type package of complementary inputs in economies with low agricultural productivity and a large share of the labor force still in agriculture. As suggested by theory, these effects are not evident in countries that already export large proportions of their agricultural production, usefully limiting for policy-makers the number of countries where support of agricultural production is likely to lead to structural transformation.

The results suggest a particularly strong role for fertilizer, which is highly consistent with field station agronomic evidence. Fertilizer's high private return on experimental plots and in the field suggests some sort of market failure. Scholars debate whether this is due to credit constraints or non-rational behavior on the part of farmers (Duflo et al., 2008, Duflo et al., 2011). Regardless, the evidence presented in this paper suggests social returns from fertilizer use that exceed the immediate private returns, furthering the case for policy efforts.

It is worth briefly describing the main concerns about increasing fertilizer use. One set is environmental. These are legitimate and require foresight in policy planning (Pingali, 2012). However, countries should not simply avoid fertilizers for environmental reasons, since soil degradation induced by fertilizer omission can pose much greater risks to agricultural production (Palm et al., 2004). A second class of concerns focuses on inequality and the potential scale bias of modern inputs. Hayami and Ruttan (1985) review the evidence on the alleged scale bias in the Asian green revolution and find that the evidence does not support that hypothesis. A third set of concerns focuses on both the challenges of governments implementing input support programs and also the challenges of exiting from them in due course. Though there is evidence that subsidy programs can be successful (Dorward and Chirwa, 2011), there is also evidence that they can be subject to elite capture, and concern that their fiscal drag effects can outlive their usefulness (e.g., Pan and Christiaensen, 2012, Pauw and Thurlow, 2014).

While our results provide some evidence for a causal link from agricultural productivity increases to structural change and higher non-agricultural labor productivity, we can only speculate on the mechanisms through which these effects play out. Nevertheless, our identification of a causal link from yield increases to labor composition shifts rules out models where structural change is driven solely by “pull” forces from growing non-agricultural sectors. To the extent that yield increases contribute to increases in non-agricultural labor productivity growth, this suggests that structural change involves more than just the satiation of food needs and the movement of labor into other sectors. This labor share shift somehow accelerates labor productivity growth. One possible channel might be increasing returns in the non-agricultural sector, perhaps through learning-by-doing . Perhaps increased food production lowers average prices and frees up consumers' resources for other consumption and for productive public and private investments, raising labor productivity elsewhere. Or perhaps higher availability of staple foods promotes health and labor productivity across sectors. Identifying more precise causal pathways between staple yields and structural change forms an important topic for future work.

## Appendix A Supplementary data

Application 1

## Footnotes

1 See Carlino and Kerr (2014) for a review of mechanisms and evidence linking agglomeration to innovation.

2 The variable for fertilizer use was redefined in 2002, when FAO's survey methodology changed and some countries shifted from a crop year to a calendar year basis. We therefore limit our analysis to 1961-2001 in order to maintain consistency. All graphs and regressions exclude more recent years accordingly.

3 The sample is more fully described in Section 4 below.

4 The index is available on the Commodity Price Data (“The Pink Sheet”). The World Bank cites the sources for the prices themselves as Fertilizer Week, Fertilizer International, and the World Bank. The price of phosphate rock is from Morocco; DAP and TSP prices are spot prices in the US Gulf; potassium chloride is from the spot price in Vancouver; and Urea prices are from the spot market in Eastern Europe (1985–1991) and then Yuzhnyy in the Black Sea (starting in 1991). Given that the prices are measured near the point of production and are f.o.b. prices, they do not include transportation costs.

5 We combine FAO fertilizer consumption data with Comtrade data United Nations (2015) to look at the proportion of fertilizer consumption in our sample countries coming from the countries we designate as fertilizer producers from the 10 companies' locations. While this does not give us information on the market share of the ten companies specifically, it shows that the places we indicate on our map as being fertilizer producers produce more than half of the fertilizer consumed in the developing world.

6 In some instances, we use exchange rates from www.oanda.com when calculated fertilizer prices are off by one or more orders of magnitude compared to other countries.

7 That is, doubling output from 30 units of 100 total to 60 units of 130 total (=100 + 30) gives an aggregate annual growth rate of 5.4 percent over 5 years.

8 Other work has found large degrees of error in national level data. For example, “in some cases a sector of the economy is known to consist of one large operator and many small ones and a qualified guess can be made as to how much of the sector is dominated by the large operators for which basic statistical data are available. The statistical office … assume[s] that the rest of the sector grows proportionally.” Sometimes, in “the subsistence economy…growth [is] assumed to take a certain value per rural household, and then assumed to grow in accordance with rural population growth.” Jerven (2010). These gross measurement errors could certainly generate severe attenuation in regression estimates.

9 Note that there is an imperfect match between the cereal yield and fertilizer variables: fertilizer use is reported as the average use over all arable land, which introduces measurement error into our specification if fertilizer use in cereals and non-cereals is not consistent. To assuage this concern, we tested the estimations below controlling for the percentage of total agriculture planted to cash crops; the cash crop variable was not significant in any of the specifications and had no effect on the point estimates discussed below.

10 All results are robust to including these countries, with one exception: Trinidad and Tobago is a very small country that is also a fertilizer producer, which makes it an outlier since its agriculture-weighted average distance to fertilizer production is orders of magnitude lower than other developing countries (even those that produce fertilizer, like India and China). Results are available upon request.

11 Calculated as follows (units in tons): $-0.00075^{\times }\;-10/9.60\,$

12 0.20=exp(0.5*0.37)−1

13 This result is robust to using lower thresholds, such as 60% growth in area harvested. It is also robust to dropping the country with highest growth in area harvested under cereals (Paraguay).

14 The result is qualitatively unchanged if we use a different threshold to categorize cereal exporters, such as 25% of production instead of 10%.

15 Results are materially unchanged using a 10-year lag, assuaging concerns of spurious 9-year correlation.

16 Polygons of natural gas fields are from the World Petroleum Assessment of the United States Geological Survey.

17 Note that while one might consider taking the log of the instrument when regressing log yields, the instrument itself is a quotient of a time-varying element (global fertilizer price) divided by a country-varying element (cost-distance). The log of the quotient is equal to the log of the numerator minus the log of the denominator, so the log of our instrument is collinear with country and time dummies.

Appendix A Supplementary data associated with this article can be found in the online version at doi:10.1016/j.jdeveco.2017.02.007.
