# Supplementary material for: Fertilizing growth: Agricultural inputs and their effects in economic development
Source: J Dev Econ. 2017 Jul;127:133–52. doi: 10.1016/j.jdeveco.2017.02.007 (PMC5726111; doi:10.1016/j.jdeveco.2017.02.007)
Supplement: Application 1 [file mmc1.pdf]

Appendix for:

Fertilizing Growth: Agricultural Inputs and their Effects in Economic Development

John W. McArthur*

Gordon C. McCord†

September 6, 2016

*jmcarthur@brookings.edu; Brookings Institution, United Nations Foundation

†gmccord@ucsd.edu; School of Global Policy and Strategy, University of California, San Diego; 9500 Gilman Drive #0519, La Jolla, CA 92093; (858) 534-0590; Fax: (858) 534-3939

Effects on Labor Productivity in Agriculture

While our analysis has studied the impact of agronomic yield increases on structural transformation, an alternative measure is to focus on labor productivity in agriculture. We consider agricultural value added per worker in agriculture, with the caveat that this represents labor productivity across the entire sector (including livestock and cash crops), and thus a measure less relevant for studying the impact of raising productivity in the staple food sector. In any case, the results remain consistent in the case of fertilizer raising agricultural labor productivity, and in turn raising GDP and reducing labor shares in agriculture. Table A.4 shows the results for labor share regressions using agricultural value added per worker instead of yields. Column I shows the OLS regression, controlling for country and year fixed effects plus total fertility rates and key macroeconomic variables. Lagged agricultural value added per worker is negatively associated to labor share, significant to the 1% level. The coefficient of -9.65 suggests that a 30% increase in agricultural labor productivity (comparable to the 30% increase in yields that a 0.5 ton increase represents above the mean) would decrease labor shares in agriculture by around 2.9 percentage points. The 2SLS approach using our instrument is shown in Columns II-III. Our instrument is negatively correlated to agricultural value added per worker, though it less statistically significant, and the F-test value of 3.71 indicates that the instrument is not strong. The second stage coefficient on yields is -25.48, suggesting that a 30% increase in value added per worker would decrease labor shares in agriculture by 7.6 percentage points (a larger effect than those shown in Table 6). Given that these results are qualitatively consistent with our results using yields per hectare, that they are testing a somewhat different concept, that the first stage is weaker, and that the specification with yields leads to a more conservative result, we maintain the specification with yields as preferred.

GMM Estimation

Fixed effects estimators suffer from dynamic panel bias particularly pertaining to bias on the lagged dependent variable (Wooldridge, 2002; Bond, 2002). A complementary estimation strategy for the economic growth equations is therefore pursued through the use of Arellano and Bond's (1991) generalized method of moments (GMM) "difference" estimator, which purges the fixed effects. The GMM strategy takes a standard first difference transform of equation (3), using lags as instruments:

$$\Delta y_{it} = \rho \Delta y_{i,t-5} + \lambda_1 \Delta a_{i,lag\,t} + \lambda_2 \Delta k_{i,t-5} + \lambda_3 \Delta r_{i,t-5} + \omega' \Delta MAC_{i,t-5} + \eta_t^a + \Delta \nu_{it}^a \quad (1)$$

Note that the first difference is taken across five-year intervals in this construction; the identification strategy holds as long as there is no autocorrelation within countries beyond the first lag. Arellano-Bond AR(2) tests are therefore applied in all GMM specifications, as are Sargan tests. For completeness, we also test the Blundell and Bond (1998) "system GMM" estimator, although it is more appropriate for random walk-type estimations and in this context may result in bias inherent in its application to cross-country regressions (Roodman, 2009). More recent work on dynamic panel estimation has employed mean group estimators that are unbiased in the presence of nonstationarity in residuals as well as unobserved cross-sectional correlation. We implement the common correlated effects mean group estimator employed in production function estimates by Eberhardt and Teal (2013).

Table A.5 presents a NAVA growth framework using GMM instrumentation and finds similar agricultural productivity effects on value added in non-agricultural sectors. Column I runs difference GMM and finds that a 9-year lag on yield is associated with subsequent increases in non-agricultural value added per worker, with a coefficient of 0.08 significant to the 10% level. Column II adds the fertilizer price instrument to the exogenous variables in the specification, and finds a coefficient on yields of 0.09 significant to the 5% level. The coefficients suggest that a 0.5 ton increase in yields leads to 4-4.5 percent higher non-agricultural labor productivity 9 years later, which translates to a 0.4-0.5 percentage point higher growth rate. Note that this magnitude lies between the fixed effects coefficients of 0.03-0.06 and the IV coefficients of 0.17-0.24 in Table 7, thus supporting the overall results. The specification in column II passes the Sargan test for overidentification of instruments with a p-value of 0.31, and AR(1) and AR(2) tests appropriately affirm first-order autocorrelation and fail to reject the null on zero second-order autocorrelation. We also employ the Blundell-Bond "system" GMM estimator, but do not report results because this does not pass a Sargan test under any relevant specification, so we prefer to interpret only the difference GMM specifications. Columns III and IV repeat these specifications using 10-year lags on yield. Coefficients on the lagged yield are now comfortably within 5% confidence levels, and their value of 0.10-0.13 implies that a 0.5 ton increase in yields leads a higher annual growth rate in non-agricultural labor productivity of 0.5-0.6 percentage points.

Conley-Hansen-Rossi Test on Exogeneity

As with the results on GDP, the higher coefficient on yields in the 2SLS framework suggests that OLS is biased downwards due to attenuation from measurement error, or due to an omitted variable that is negatively correlated to yields and to slower labor movement out of agriculture (such as distortionary policies or extensification by farmers, as discussed earlier). Given that the results on labor share are our preferred evidence for the causal effects of yield increases on structural transformation, we go beyond the controls

in columns IX-X in exploring the possibility of an exclusion restriction violation. Conley, Hansen, and Rossi (2012) develops methods for performing inference while relaxing the exclusion restriction; specifically, the method derives 95% confidence intervals for the second stage coefficient on the endogenous variable if the instrument were included in the second stage and had a nonzero coefficient (violating the exclusion restriction). In the case of the labor share regression, the maximum coefficient on the instrument is 0.015, above which the 95% confidence interval for the coefficient on yields includes zero. Since the standard deviation of agricultural labor share in the sample is 21.8 and the standard deviation of the instrument is 33.95, then the coefficient of 0.016 would mean that a one standard deviation in the instrument would lead to a $.025\sigma$ change in labor share. This compares to the effect through yields, where a 1σ change in the instrument leads to a 0.6σ change in labor shares. The results in Table 6 are therefore robust to a small relaxation of the exclusion restriction. Running the same test on the results on GDP leads to stronger evidence of robustness: a one standard deviation change in the instrument would have to lead to more than a 0.2σ change in income per capita through a channel other than yields in order to reduce the coefficient on yield to zero. This compares to the effect of yields on income in 2SLS, whereby a one standard deviation in yield leads to 0.3σ change in income. Negating our results would therefore require that most of the effect of fertilizer price changes on income occur through a channel other than yields, which is highly unlikely.

We also run the Conley, Hansen, and Rossi (2012) test on the regressions of yield on fertilizer, using our instrument in 2SLS, and find that our results are robust to significant violations of exclusion restriction. Specifically, we find that the coefficient on the instrument in a second stage (the violation of the exclusion restriction) would have to be to be equal to or greater than 0.01 in order for the coefficient on fertilizer to lose statistical significance. Since the standard deviation of yields in the data is 1.02 and the standard deviation of the instrument is 28.6, then a 0.01 coefficient would mean that a one standard deviation in the instrument would lead to a 0.28σ change in yields. This compares to the effect through fertilizers, where a 1σ change in the instrument leads to a 0.25σ change in yields using the coefficients from the first and second stage regressions in Table 3. We conclude that the violation of the exclusion restriction would have to be as large as the effect measured through fertilizer in order for it to negate our results. This is unlikely since at least some of the effect of the instrument on yields is presumably working through fertilizer use.

Supplementary Figures

Figure A.1 compares the growth of cereal yields to growth in GDP per capita over the 1965 to 2001 period, exhibiting a positive correlation of 0.44.

Figure A.1: Growth in GDP per capita vs. Growth in Cereal Yields, 1965-2001

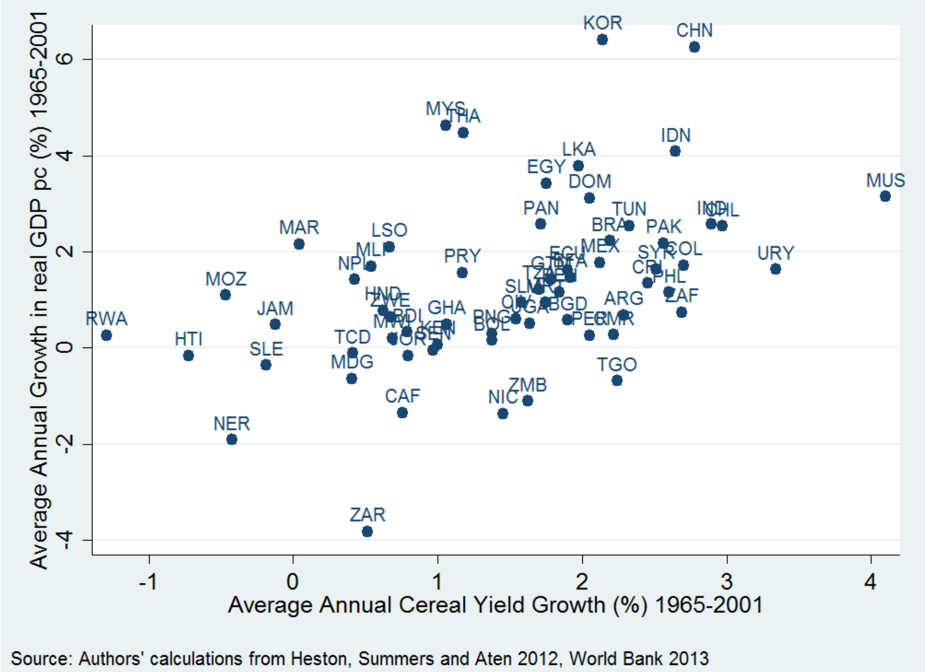

A novel relationship is presented in Figure A.2 , which compares 1965 cereal yield levels to subsequent GDP growth from 1965-2001 across developing countries, excluding fuel exporters and socialist economies. The horizontal line marks zero average growth and the vertical line marks 2 t/ha of cereal yields. In addition to the overall positive relationship between initial yield and economic growth, it is noteworthy that no country in the sample experienced negative average growth after reaching a yield threshold of 2 t/ha. The threshold is only marginally affected by the inclusion of fuel exporting and socialist economies, with only Cuba, North Korea and Venezuela falling just under the vertical line in the lower-right quadrant of the figure. Romania and Saudi Arabia fall in a similar location on the graph if the sample is further expanded to include Europe and higher income countries.

Figure A.2: Growth in GDP per capita vs. Initial Cereal Yields, 1965-2001

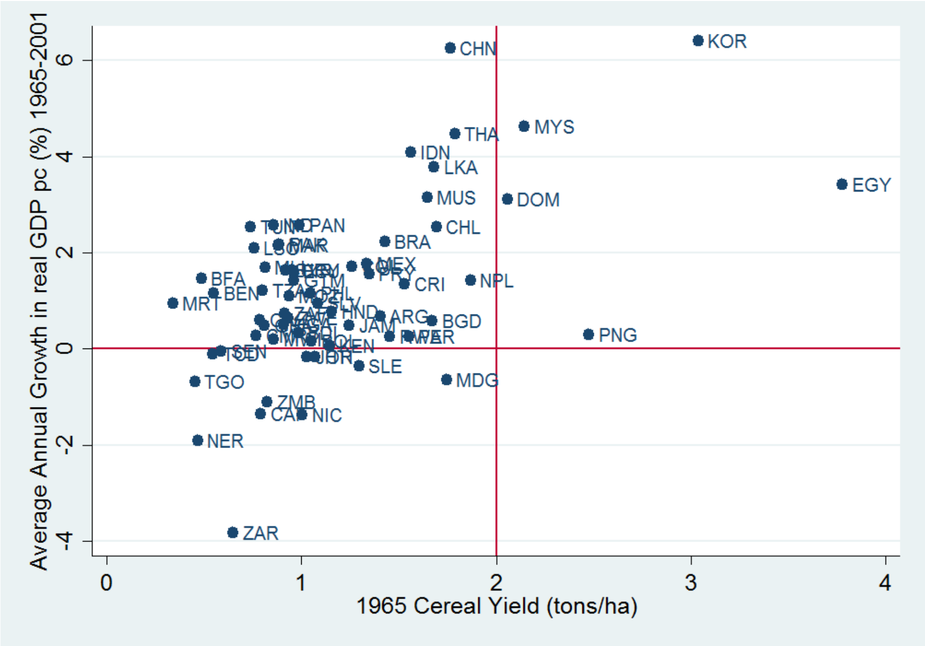

Source: Authors' calculations from Heston, Summers and Aten 2012, World Bank 2013

Supplementary Tables

Table A.1: Major Fertilizer Company and Geolocated Production Locations

Company	Location	Latitude	Longitude
Agrium	Alberta, Canada	54.500	-115.000
	Saskatchewan, Canada	54.500	-105.680
	Washington, USA	47.500	-120.500
	California, USA	37.000	-120.000
	Idaho, USA	45.000	-114.000
	Texas, USA	31.000	-100.000
	Bahia Blanca, Argentina	-38.720	-62.270
	Alexandria, Egypt	31.198	29.919
CF Industries	Courtright, ON, Canada	42.780	-82.350
	Donaldsonville, LA, USA	30.100	-90.994
	Medicine Hat, AB, Canada,	50.042	-110.678
	Port Neal, IA, USA	42.402	-96.358
	Woodward, OK, USA	36.433	-99.398
	Yazoo City, MS, USA	32.856	-90.408
EuroChem	Novomoskovskiy Azot, Russia	54.033	38.267
	Nevinnomysskkiy Azot, Russia	44.633	41.933
	Antwerp, Belgium	51.217	4.400
Indian Farmers Fertilizer Cooperative Limited (IFFCO)	Kalol, Gujarat, India	22.606	73.463
	Kandla, Gujarat, India	23.030	70.220
	Phulpur, Uttar Pradesh, India	25.550	82.100
	Aonla, India	28.280	79.150
	Paradeep, Orissa, India	20.320	86.620
Koch	Oklahoma, USA	35.500	-98.000
	Kansas, USA	38.500	-98.000
	Nebraska, USA	41.500	-100.000
	Iowa, USA	42.000	-93.000
	Manitoba, Canada	55.067	-97.517
Orascom Construction Industries	Geleen, Netherlands	50.967	5.833
Potash Corporation of Saskatchewan	Augusta, GA, USA	33.470	-81.975
	Geismar, LA, USA	30.204	-91.022
	Lima, OH, USA	40.741	-84.115
	Point Lisas, Trinidad & Tobago	10.391	-61.474
Sinopec	Hubei Province, China	31.200	112.300
	Janling/Nanjing, China	32.050	118.767
	Lanzhou, Gansu, China	36.050	103.800
	Anqing, China	30.500	117.033
	Guangzhou, China	23.133	113.267
	Zhenhai, China	29.960	121.720
	Urumqi, China	43.825	87.600
	Ningxia, China	38.467	106.267
	Dalian, China	38.921	121.639
	Daqing, China	46.583	125.000
	Dongting, China	29.317	112.950
TogliattiAzot	Tolyatti, Russia	53.509	49.422
Yara International	Yara Belle Plaine, Saskatchewan,	50.455	-104.607
	Rio Grande, Brazil	-32.035	-52.099
	Trinidad, Trinidad & Tobago	10.461	-61.249
	Montoir, France	47.329	-2.148
	Pardies, Fance	43.367	-0.585
	Ambes, France	45.012	-0.539
	Ribecourt, France	49.511	2.923
	Le Havre, France	49.490	0.100
	Sluiskil, Netherlands	51.278	3.838
	Tertre, Belgium	50.483	3.832
	Brunsbuttel, Germany	53.896	9.139
	Rostock, Germany	54.083	12.133
	Porsgrunn, Norway	59.116	9.710
	Koping, Sweden	59.517	15.983
	Glomfjord, Norway	66.816	13.944
	Uusikaupunki, Finland	60.800	21.417
	Ferrara, Italy	44.833	11.617
	Ravenna, Italy	44.417	12.200
	Yara Pilbara, Australia	-20.581	116.808
	Lifeco, Libya	30.435	19.667

Table A.2:

Variable	Description	Source
Fertilizer (t)	Average fertilizer use per hectare in kilograms, averaged over 3 years (t-1, t, t+1)	World Bank 2006
GDP per capita (t)	Natural logarithm of real per capita GDP in USD 2000, averaged over 3 years (t-1, t, t+1)	Penn World Tables 7.1, Heston et al. (2012)
Government Consumption (t)	Government Consumption as % of GDP (t-5 - t-1)	World Bank 2013
Inflation (t)	Average inflation (t-5 - t-1)	World Bank 2013
Investment (t)	Fixed capital formation as % of GDP averaged over 5 years (t-5 - t-1).	World Bank 2013
Irrigation (t)	Percent of cropland irrigated, averaged over 3 years (t-1, t, t+1)	World Bank 2006
Labor : land ratio (t)	Natural logarithm of agricultural labor force divided by land planted to cereals (i.e., persons/ha) at time t	World Bank 2013, FAOSTAT 2007, authors' calculations
Labor Share in Agriculture (t)	Agricultural labor force divided by total labor force, averaged over 3 years (t-1, t, t+1)	FAOSTAT 2007
Modern seeds (t)	Percent of crops planted with modern variety seeds at time t	Evenson 2006; presented in Conley et al. (2007)
Non-agricultural value added per worker (t)	Natural logarithm of value added in non-agricultural sectors (in constant $2000) divided by economically active population in non-agricultural sectors at time t	World Bank 2006, FAOSTAT 2007, authors' calculations
Population (t)	Total population at time t	World Bank 2013
Precipitation (t)	Natural logarithm of annual precipitation in millimeters, averaged over 3 years (t-1, t, t+1)	Matsuura and Willmott (2012)
Total Fertility Rate (t)	Total fertility rate, live births per woman, averaged over 3 years (t-1, t, t+1)	World Bank 2013
Tractors (t)	Tractors per hectare, averaged over 3 years (t-1, t, t+1)	World Bank 2013
Years schooling (t)	Total years of schooling at time t	Barro-Lee 2013
Yield (t)	Cereal yield per hectare in kilograms, averaged over 3 years (t-1, t, t+1).	World Bank 2006

Table A.3: 75-Country Sample

Argentina	Liberia^
Bangladesh	Madagascar
Benin	Malawi
Bolivia	Malaysia
Brazil	Mali
Burkina Faso	Mauritania
Burundi	Mexico
Cambodia	Mongolia
Cameroon	Morocco
Central African Republic	Mozambique
Chad^	Myanmar^
Chile^	Nepal
China	Nicaragua^
Colombia	Niger^
Congo, Dem. Rep.	Pakistan
Costa Rica	Panama
Cote d'Ivoire	Papua New Guinea
Cuba^	Paraguay
Dominican Republic	Peru
Ecuador	Philippines
Egypt, Arab Rep.	Rwanda^
El Salvador	Senegal
Eritrea^	Sierra Leone^
Ethiopia^	South Africa
Ghana	Sri Lanka
Guatemala	Sudan
Haiti^	Syrian Arab Republic^
Honduras	Tanzania
India	Thailand
Indonesia	Togo
Jamaica	Tunisia
Jordan	Uganda
Kenya	Uruguay
Korea, Dem. Rep.^	Vietnam^
Korea, Rep.	Yemen, Rep.
Lao PDR^	Zambia
Lebanon^	Zimbabwe
Lesotho	

^ These 17 countries are not in the 58-country sample for GDP, Labor Share, and NAVA regressions

Table A.4:

	Dependent Variable		
	**Labor Share in Agriculture**	**ln (Agricultural Value Added per Worker)**	**Labor Share in Agriculture**
	(I)	(II)	(III)
Independent variables		2SLS	
5-year lag in Global Fert Price / ln(Cost-Adjusted Distance to Nitrogen Production Site)		-0.0020*	
		(0.0010)	
5-year lag ln(Agricultural Value Added per Worke	-9.65***		-25.48***
	(2.19)		(8.32)
Ave. Investment (t-5 to t-1)	-0.04	0.001	-0.02
	(0.05)	(0.005)	(0.10)
ln(Inflation (t-5 to t-1))	-0.23	-0.04*	-0.90
	(0.36)	(0.02)	(0.63)
Gov't Consumption as % of GDP (t-5 to t-1)	0.20**	-0.005	0.11
	(0.10)	(0.006)	(0.16)
Total Fertility Rate (t-5)	2.36***	-0.04	1.75*
	(0.64)	(0.03)	(0.95)
N	262	262	
Countries	58	58	
Within R-squared	0.86	0.42	0.75
Kleibergen Paap F Test on First Stage		3.71	
Country Dummies	Y	Y	Y
Year Dummies	Y	Y	Y

Notes: Standard errors in parentheses, clustered by country in both first and second stages. *, **, and *** represent 10%, 5%, and 1% significance levels, respectively. All variables are 3 year means measured at 5 year intervals. E.g., "1970" measures means over 1969, 1970 and 1971. The subsequent value averages over 1974, 1975 and 1976. Constant terms, year dummies, and country dummies not reported to save space.

Table A.5:

	Dependent Variable			
	**ln (non agriculture value added per worker)**	**ln (non agriculture value added per worker)**	**ln (non agriculture value added per worker)**	**ln (non agriculture value added per worker)**
	(I)	(II)	(III)	(IV)
Independent variables	Difference GMM	Difference GMM w/IV	Difference GMM	Difference GMM w/IV
5-year lag ln(non ag value per worker)	0.68***	0.68***	0.63***	0.65***
	(0.10)	(0.11)	(0.09)	(0.10)
9-year lag yield	0.08*	0.09**		
	(0.04)	(0.04)		
10-year lag yield			0.10**	0.13***
			(0.05)	(0.04)
Ave. Investment (t-5 to t-1)	0.01***	0.01***	0.01***	0.01***
	(0.004)	(0.003)	(0.003)	(0.004)
ln(Inflation (t-5 to t-1))	-0.07	-0.07	-0.06	-0.07*
	(0.04)	(0.05)	(0.05)	(0.04)
Gov't Consumption as % of GDP (t-5 to t-1)	-0.007	-0.008	-0.005	-0.007
	(0.006)	(0.006)	(0.007)	(0.006)
Total Fertility Rate (t)	0.02	0.02	0.02	0.03
	(0.06)	(0.06)	(0.06)	(0.06)
N	201	201	201	201
Countries	53	53	53	53
Arellano-Bond test for AR(1)	0.04	0.04	0.09	0.08
Arellano-Bond test for AR(2)	0.16	0.18	0.16	0.19
Sargan Test p-value	0.31	0.31	0.36	0.36

Notes: Robust standard errors in parentheses. *, **, and *** represent 10%, 5%, and 1% significance levels, respectively. All variables are 3 year means measured at 5 year intervals. E.g., "1970" measures means over 1969, 1970 and 1971. The subsequent value averages over 1974, 1975 and 1976. Constant terms, year dummies, and country dummies not reported to save space.

References

Arellano, Manuel and Stephen R. Bond. 1991. "Some Tests of Specification for Panel Data: Monte Carlo Evidence and an Application to Employment Equations." *Review of Economic and Studies* 58 (2):277–297.

Blundell, Richard and Stephen R. Bond. 1998. "Initial conditions and moment restrictions in dynamic panel data models." *Journal of Econometrics* 87:11–143.

Bond, Stephen R. 2002. "Dynamic Panel Data Models: A Guide to Micro Data Methods and Practice." Tech. rep., Institute for Fiscal Studies.

Conley, Timothy G., Christian B. Hansen, and Peter E. Rossi. 2012. "Plausibly Exogenous." *Review of Economics and Statistics* 94 (1):260–272.

Eberhardt, Markus and Francis Teal. 2013. "No Mangoes in the Tundra: Spatial Heterogeneity in Agricultural Productivity Analysis." *Oxford Bulletin of Economics and Statistics* 75 (6):914–939.

Roodman, David. 2009. "A Note on the Theme of Too Many Instruments." *Oxford Bulletin of Economics and Statistics* 71 (1):135–158.

Wooldridge, Jeffrey M. 2002. *Econometric Analysis of Cross Section and Panel Data.* Cambridge, MA: MIT Press.
